# Synthesis and Primary Activity Assay of Novel Benitrobenrazide and Benserazide Derivatives

**DOI:** 10.3390/molecules29030629

**Published:** 2024-01-29

**Authors:** Karolina Juszczak, Wojciech Szczepankiewicz, Krzysztof Walczak

**Affiliations:** Department of Organic Chemistry, Bioorganic Chemistry and Biotechnology, Faculty of Chemistry, Silesian University of Technology, 44-100 Gliwice, Poland; karolina.juszczak@polsl.pl (K.J.); wojciech.szczepankiewicz@polsl.pl (W.S.)

**Keywords:** Schiff bases, benitrobenrazide, benserazide, hexokinase 2, enzyme inhibition

## Abstract

Schiff bases attract research interest due to their applications in chemical synthesis and medicinal chemistry. In recent years, benitrobenrazide and benserazide containing imine moiety have been synthesized and characterized as promising inhibitors of hexokinase 2 (HK2), an enzyme overexpressed in most cancer cells. Benserazide and benitrobenrazide possess a common structural fragment, a 2,3,4-trihydroxybenzaldehyde moiety connected through a hydrazone or hydrazine linker acylated on an N′ nitrogen atom by serine or a 4-nitrobenzoic acid fragment. To avoid the presence of a toxicophoric nitro group in the benitrobenrazide molecule, we introduced common pharmacophores such as 4-fluorophenyl or 4-aminophenyl substituents. Modification of benserazide requires the introduction of other endogenous amino acids instead of serine. Herein, we report the synthesis of benitrobenrazide and benserazide analogues and preliminary results of inhibitory activity against HK2 evoked by these structural changes. The derivatives contain a fluorine atom or amino group instead of a nitro group in BNB and exhibit the most potent inhibitory effects against HK2 at a concentration of 1 µM, with HK2 inhibition rates of 60% and 54%, respectively.

## 1. Introduction

Carbonyl compounds easily react with N-centered nucleophiles, such as amines, hydrazine, and its derivatives. Schiff bases, a product of condensation of carbonyl compounds with primary amines, have a wide range of applications in pharmaceuticals [1,2,3] and in coordination chemistry as ligands and chelating agents [4,5]. The versatile pharmacophore C=N is present in biologically active compounds exhibiting antioxidant [3,6], antimicrobial [3,6,7], and anticancer [8] properties.

**Figure 1:**
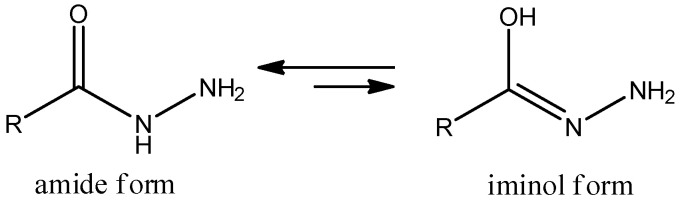
Tautomerism in a hydrazide molecule.

N-Acylhydrazides R(CO)NHNH_2_ are structurally like amides and a relocation of a proton can occur, thus forming an iminol form (Figure 1) [1]. Compounds containing an amide–imine bridge -C(=O)-NH-N=CH- can be considered hybrid structures of hydrazide or hydrazones [1]. These hybrid compounds have several applications in pharmacology, exhibiting antimicrobial activity [9,10,11], anticancer activity [8,9], and free radical scavenging properties [12].

**Figure 2:**
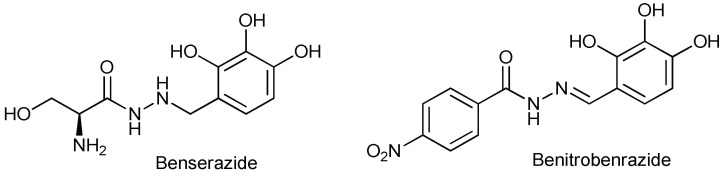
The chemical structures of reported HK2 inhibitors.

Benserazide (BND) and benitrobenrazide (BNB) (Figure 2) are compounds belonging to the group of hydrazine derivatives containing common structural fragments, including a N-acyl rest of serine or 4-nitrobenzoic acid and a 2,3,4-trihydroxybenzaldehyde (pyrogallol-4-carboxaldehyde) fragment attached to an N′ nitrogen atom by a methylene or methine carbon atom. Both compounds BNB and BND have promising inhibitory activity against hexokinase 2 (HK2) [13,14,15], an enzyme involved in the phosphorylation of glucose in glycolysis, a first step of glucose metabolism [16]. An increased requirement for glucose is observed in cancer cells, especially in rapidly growing and drug-resistant tumors [17,18,19,20,21,22]. Among all human hexokinase isoenzymes, HK2 shows overexpression in cancer cells, which makes it interesting in the context of molecularly targeted therapy [23,24,25,26,27,28]. Currently, several HK2 potent inhibitors are recognized, like metformin, 2-deoxy-D-Glucose, or 3-bromopyruvate [24,29,30,31,32].

Benserazide is an FDA-approved drug for the treatment of Parkinson’s disease, but it was recently also recognized as a strong HK2 inhibitor [14]. Benitrobenrazide was identified as a potential HK2 inhibitor by structure-based virtual ligand screening [15]. According to our previous study, the key structural feature is the presence of three hydroxy groups in a benzene ring, which occupies the same HK2-binding pocket as the natural substrate, glucose [29]. The influence of other structural elements, namely -NH-NH-CH_2_- and -NH=N-CH-, on HK2 enzymatic activity has not yet been determined and requires investigation to explain their impact on enzymatic activity.

Herein, we present our primary attempts to explore the influence of structural features observed in benserazide and benitrobenrazide in their biological activity against HK2. The serine originally present in benserazide has been exchanged with other amino acid fragments, e.g., glycine, tyrosine, and cysteine. Application of these amino acids that are different in structure, including glycine (achiral), threonine-containing benzene ring, and cysteine (containing a thiol group instead of a hydroxyl one), in the synthesis of benserazide analogues should deliver an answer regarding the importance of serine moiety on benserazide inhibitory activity. In the BNB molecule, the 4-nitrophenyl moiety has been substituted by an alkyl chain or aromatic rings of different molecular areas (benzene, naphthalene, and anthracene) to judge their downstream influence on HK2 activity and gauge HK2 active site volume. We decided to exchange the toxicophoric nitro group present in BNB moiety by two common substituents, namely the fluorine atom and the amino group [33,34]. These substituents have the opposite effects on electron density in the benzene ring of BNB and were introduced to explain the eventual influence of electronic conditions on its activity. The fluorine atom, like the nitro group, decreases the electron density, whereas the amino group is typical of the electron-donating group. The novel analogues of benserazide and benitrobenrazide were examined for their inhibitory activity against HK2 to obtain information about the influence of structural variations on inhibition activity.

## 2. Results and Discussion

### 2.1. Chemistry of Benitrobenrazide and Benserazide Derivatives

Benitrobenrazide and benserazide contain a *N*-acyl fragment, 4-nitrophenyl, or a serine moiety, respectively [13,15]. We modified these regions of each molecule by incorporating aromatic rings of different molecular areas instead to probe the volume of the active site pocket of HK2. In further modifications, a nitro group suspected as a genotoxic substituent was replaced by substituents of different polarity, constituting either a fluorine atom or an amino group. The introduction of fluorine and amino substituent as pharmacophore groups into drugs molecules increases their therapeutic effectiveness [35,36]. Additionally, the imine bond in hydrazone analogue was reduced to a single carbon–nitrogen bond to identify the effect of the imine double bond (C=N) on HK2 activity.

**Scheme 1:**
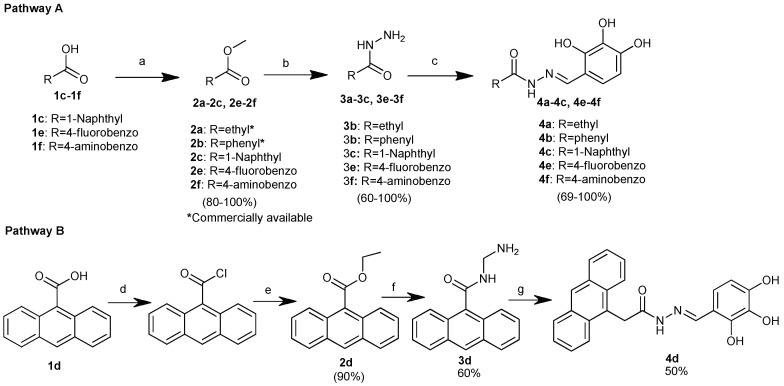
Preparation of benitrobenrazide derivatives. Reagent and conditions: (A) **a**: SOCl_2_ (1.2 equiv.), MeOH, 0 °C to RT, 24 h; **b**: N_2_H_4_·H_2_O (98%, 4 equiv.), MeOH, RT/reflux 24/72 h.; **c**: (HO)_3_C_6_H_2_CHO; (1.0 equiv.), MeOH, RT/reflux, 24 h. (B) **d**: SOCl_2_, (53.6 equiv.), DMF, RT, Ar, 4 h; **e**: EtOH, TEA (1.2 equiv.), RT, 24 h; **g**: N_2_H_4_·H_2_O (98%, 100 equiv.), reflux, 72 h; **g**: (HO)_3_C_6_H_2_CHO; (1.1 equiv.), MeOH, reflux; 24 h.

The final benitrobenrazide analogue synthesis is depicted below (Scheme 1). Commercially available methyl propionate and benzoate **2a** and **2b** were used. Methyl esters of other aromatic acids **1c**, **1e**, and **1f** were obtained in a one-pot synthesis involving the primary transformation of each appropriate carboxylic acid into its respective acyl chloride by treatment with thionyl chloride in excess, followed by esterification in the presence of methanol (MeOH) [37] (Scheme 1; Pathway A). The ester of anthracene-9-carboxylic acid **1d** was synthesized by a two-step synthesis. The first step was conversion of anthracene-9-carboxylic acid into its acyl chloride in the presence of an excess of thionyl chloride and a small amount of dimethylformamide (DMF) as a catalyst. The separated acyl chloride was then subjected to esterification with ethanol (EtOH) in the presence of triethylamine, according to synthesis pathway B shown in Scheme 1.

Hydrazides **3a**–**3f** were obtained with a good yield from appropriate esters in nucleophilic substitution on a carbonyl carbon atom in the presence of an excess of hydrazine monohydrate (four equivalents) in anhydrous boiling methanol [38,39]. Conducting hydrazide synthesis **3b**–**3c** and **3f** at room temperature led to a purer product and a good yield (60–77%). The preparation of hydrazide **3d** needs harsher conditions, so the reaction was conducted in an excess of boiling hydrazine hydrate, with a prolonged reaction time (Scheme 1; B pathway). Hydrazides **3b**–**3f** were obtained in the form of white solids, which precipitated during the reaction process and crystallized from ethanol or aqueous ethanol. Compound **3a** was purified via silica gel column chromatography using MeOH/CHCl_3_ (1:1 *v*/*v*) as an eluent**.** The hydrazide structure was confirmed by ^1^H NMR spectroscopy. The absence of a singlet assigned to the methyl group in the region of 3.25–3.95 ppm confirmed the conversion of the esters into hydrazides.

**Figure 3:**
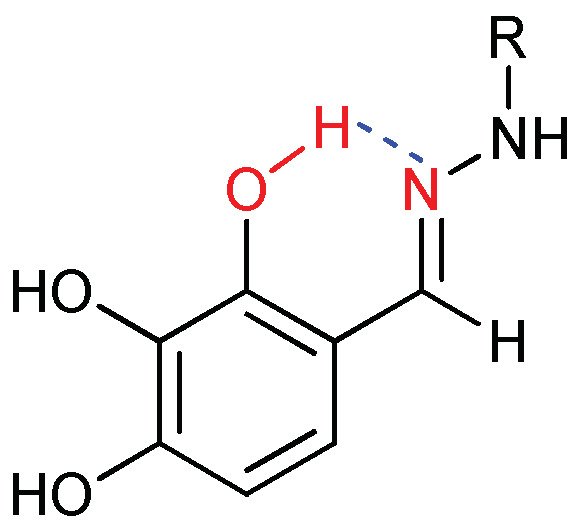
Intramolecular hydrogen bonding interaction in synthesized Schiff base **4**.

Compounds detailed in **3**, treated with 2,3,4-trihydroxybenzaldehyde in methanol, produced the products listed in **4** at a 50–100% yield. Hydrazones **4** can adopt an E or a Z configuration at the imine double bond (-N=CH-). In the case of the E conformation, the geometrical isomer can be stabilized by the formation of an intramolecular hydrogen bond between the 2-OH hydroxyl group and the nitrogen atom of the imine [40], as is depicted in Figure 3. Similar behavior is exhibited by compound **10**.

Using quantum chemical calculations based on Density Function Theory (DFT), we studied the possibility of the existence of equilibrium between conformers E and Z for **4a**. For conformer E, the corresponding hydrogen bond length is 2.1 Å. Hydrogen bond formation confirms our DFT calculation of stretching vibrations for the O-H group at 3224.47 cm^−1^. Additionally, our calculation data show higher stability of the E conformer over the Z conformer by 9 kcal/mol when hydrogen bond formation is included in simulations [41]. We can assume that the E conformer is the predominant form of hydrazone **4a.** ^1^H NMR spectroscopy verified the results of our calculation based on DFT. The chemical shifts of the protons of the 3-OH and 4-OH groups create singlet peaks at δ H 8.4–9.6 ppm, while the peak for the proton of the 2-OH group is shifted to a lower field and was observed in the region of δ H 11.3–12.4 ppm. We attribute this down-field shift of the 2-OH group to the formation of an intramolecular hydrogen bond.

**Scheme 2:**
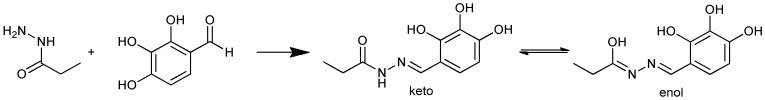
Tautomeric equilibrium in **4a** benitrobenrazide.

Based on ^1^H and ^13^C NMR spectra of **4a** in DMSO, we observed separate chemical signals for the imine proton (N=CH) at 8.09 and 8.17 ppm, respectively. In contrast, the protons of the ethyl group (CH_2_CH_3_) were present as two triplets in the region of 1.05–1.09 ppm and two quartets in the region of 2.19–2.23 and 2.51–2.54. In the ^13^C NMR spectrum, the carbon atom of the amide carbonyl groups (-C(O)NHN-) was detected from signals at 173.71 and 168.73 ppm. The signals for carbon from the ethyl groups (CH_2_CH_3_) were present at 8.47, 9.41 ppm, 25.24 and 26.97. These spectra suggest the existence of another structural feature for compounds **4**, namely a keto-enol tautomerism (Scheme 2).

Quantum chemical calculations using DFT were used to quantify Gibbs free energy for the constitutional isomers of **4a**, which confirms the possibility of the keto and enol forms’ occurrence. According to the calculations performed on data collected without a solvent, the keto tautomer is preferred over the enol tautomer and is thermodynamically more stable than the enol form by about 11 kcal/mol. However, based on calculations performed in the presence of DMSO, the solvation process changed the Gibbs free energy of both tautomers. Our calculations clearly indicate that, in a polar aprotic solvent like DMSO, the enol form is more stable by 5 kcal/mol when compared with the keto form due to the interaction of the OH-enol group with the oxygen atom of dimethyl sulfoxide (DMSO).

**Scheme 3:**
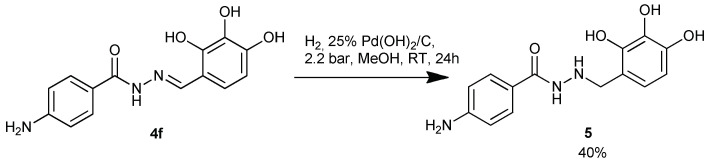
Reduction of hydrazone **4f**.

The imine bond in derivative **4f** was reduced using hydrogen in the presence of Pd(OH)_2_ as a catalyst at elevated pressure (Scheme 3). The reaction progress was monitored by ^1^HNMR spectrometry. The disappearance of the signal for the 8.39 ppm imine proton region (N=CH) indicated the consumption of substrate **4f.**

**Scheme 4:**
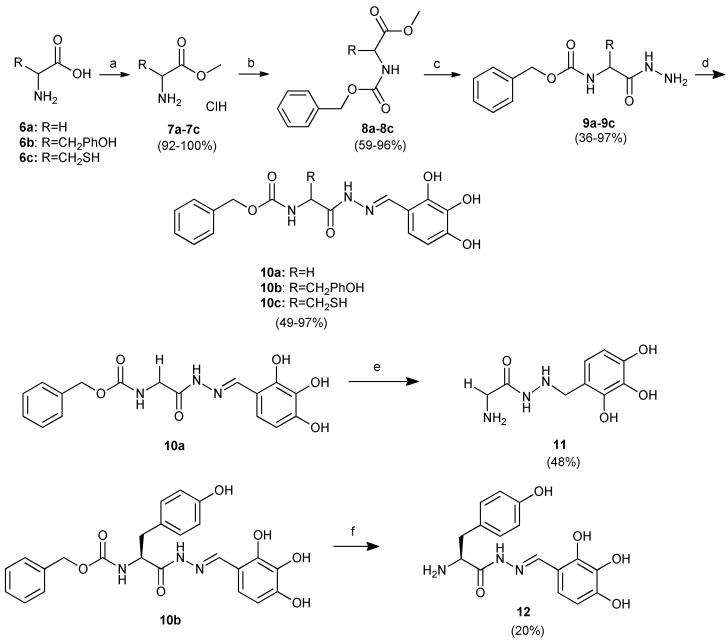
Preparation of benserazide analogues. Reagent and conditions: **a**: MeOH, SOCl_2_ (1.2 equiv.), 0 °C to RT, 24 h; **b**: CbzCl (1.2 equiv.), TEA (2.5 equiv.), DCM, RT, 24 h; **c**: 98% N_2_H_4_.H_2_O (4.0 equiv.), MeOH, RT, 24 h; **d**: (HO)_3_C_6_H_2_CHO, (1.2 equiv.), MeOH, RT, 24 h/(HO)_3_C_6_H_2_CHO; (1.2 equiv.), THF, reflux, 72 h; **e**: HCOONH_4_, 25% Pd(OH)_2_/C, MeOH, 50 °C, Ar, 12 h; **f**: H_2_, 25% mixture Pd(OH)_2_/C and Pd/C, 2.5 bar, MeOH, RT, 6 h.

Continuing our study, we synthesized benserazide analogues, in which serine was replaced with a side chain moiety of another L-amino acid glycine, tyrosine, and cysteine (Scheme 4).

Commercially available L-amino acids **6a**–**c** were converted to methyl esters **7a**–**c**, using thionyl chloride and methanol. A benzyloxycarbonyl group (Cbz) was used to protect the amino group of the amino acids by reacting esters **7** with benzyl chloroformate in the presence of triethylamine (TEA) in an anhydrous methylene chloride solution [42]. This method of protection was used to facilitate the parallel deprotection of Cbz together with catalytic hydrogenation of the double bond. The reaction of *N*-Cbz-L-amino acid esters **8a**–**c** with 98% hydrazine hydrate under the same conditions described in Scheme 4 provided products **9** as white crystals at a 36–97% yield. Hydrazones **10a** and **10c** were synthesized via the reaction of hydrazide **9a** and **9c** with 2,3,4-trihydroxybenzaldehyde in methanol at room temperature. In the case of **10b**, as the above procedure failed, we repeated the condensation in tetrahydrofuran (THF) instead of methanol in an inert atmosphere of argon at reflux, which produced product **10b** at a 55% yield. All hydrazones **10a**–**c** were obtained with the *E* configuration, as confirmed by NMR spectroscopy. The best purification method for compounds **10** was crystallization with aqueous methanol (1:1 *v*/*v*). The last step in this synthesis was the elucidation of an efficient method for the reduction of the imine bond of hydrazone and deprotection of the Cbz group. For the corresponding hydrazone **10b**, hydrogenation was performed in a Parr’s autoclave with gaseous H_2_ at 2.5 bar in the presence of palladium catalysts (mixture of 25% Pd/C and Pd(OH)_2_) at room temperature in methanolic solution. We observed only cleavage of the Cbz group.

Compound **10a** was reduced under modified conditions. Instead of gaseous hydrogen, ammonium formate was used as a hydrogen donor and the same palladium catalysts were used, according to the reported protocol [43]. Product **11** was obtained as a hygroscopic powder, which rapidly liquefied after 5 min on air. For this reason, hydrazide **11** was lyophilized after the purification was complete. In the case of the cysteine analogue of benserazide **10c**, hydrogenation did not occur under these conditions, or with the use of other catalysts such as Raney nickel and electrochemical reduction. The likely reason is that a compound containing sulfur in its structure causes catalyst poisoning, resulting in the loss of catalyst function [44].

### 2.2. Inhibitory Effect of Benserazide and Benitrobenrazide Derivatives on HK2 Enzymatic Activity

**Figure 4:**
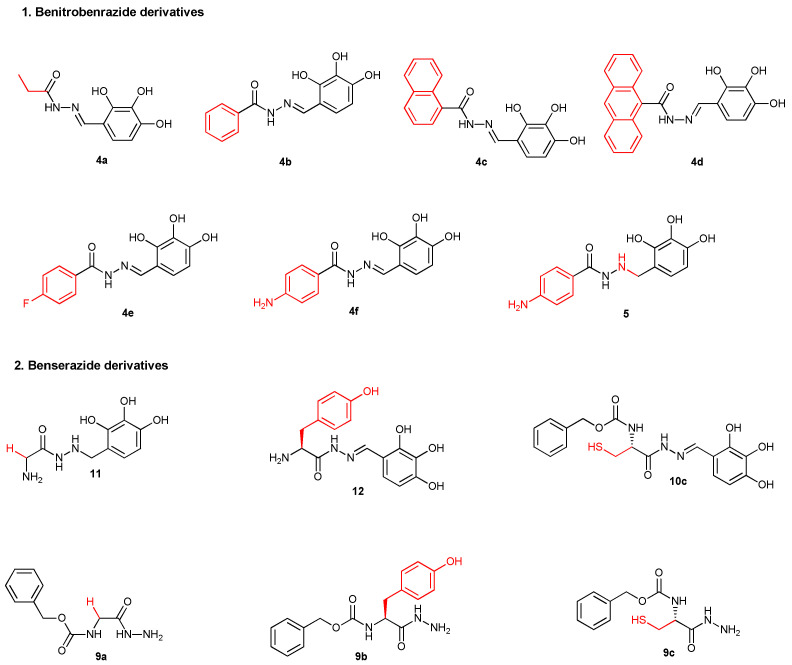
Structure of the final derivatives evaluated for inhibition of HK2.

We conducted in vitro studies of representative derivatives, namely N-acylhydrazones **4a**–**4f** as benitrobenrazide derivatives and a benitrobenrazide derivative with a single carbon-nitrogen bond **5**. The benserazide derivatives, hydrazones **10**, **12** with imine bonds, and hydrazide **11** with a single carbon–nitrogen bond, which, like benserazide, have an L-amino acid fragment (Figure 4), were selected for in vitro experiments. The selected intermediates of hydrazide **9a**–**c**, the potential peptidomimetics, were also used to evaluate any inhibition of enzyme activity (Figure 4). The chosen compounds subjected to this enzyme activity assay were purified by preparative HPLC.

For the inhibitory activity estimation, we used a colorimetric method. The most popular HK2 enzymatic activity assays reported in the literature are tests based on spectroscopy, measuring/detecting the changes in NADPH absorbance at 320–490 nm [13,14,15,31]. An alternative assay to determine the HK2 activity by reverse-phase high-performance liquid chromatography (RP-HPLC) was reported by Guan et al. [45]. According to this method, the concentration of released ADP during the conversion of glucose into 6-glucose phosphate is measured at 254 nm. We excluded RP-HPLC as an alternative assay because tested compounds contain chromophores exhibiting absorption in UV-Vis in a region of 200–250 nm, which renders the assay results unreliable. To evaluate the potential of hexokinase 2 inhibitors, we used a commercially available Hexokinase II Inhibitor Screening Kit, which uses a spectrophotometric method by measurement of absorbance at 450 nm, where tested compounds exhibit no interference with the assay. This HK2 activity assay is based on HK2’s ability to convert glucose into glucose-6-phosphate. Glucose-6-phosphate is oxidized by glucose-6-phosphate dehydrogenase to produce NADPH, which reduces the probe, showing strong absorbance at 450 nm.

**Figure 5:**
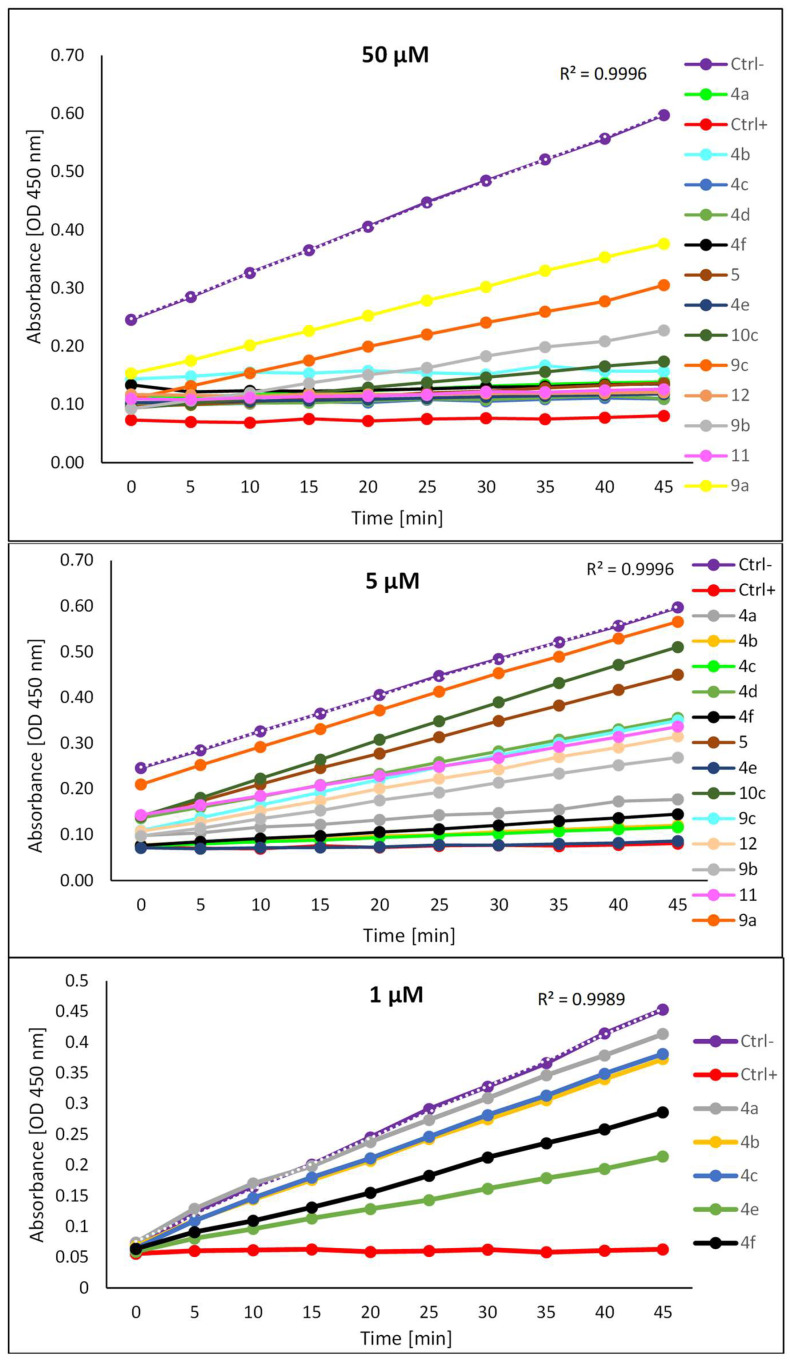
Inhibition of HK2 activity, demonstrated as a reduction in rate or extent of generation of HK2-dependent absorbance (OD) at 450 nm by a synthesized compound. Ctrl- is an enzyme control without inhibitor, which shows the normal rate of phosphorylation by HK2. All experiments were performed independently in triplicate.

**Figure 6:**
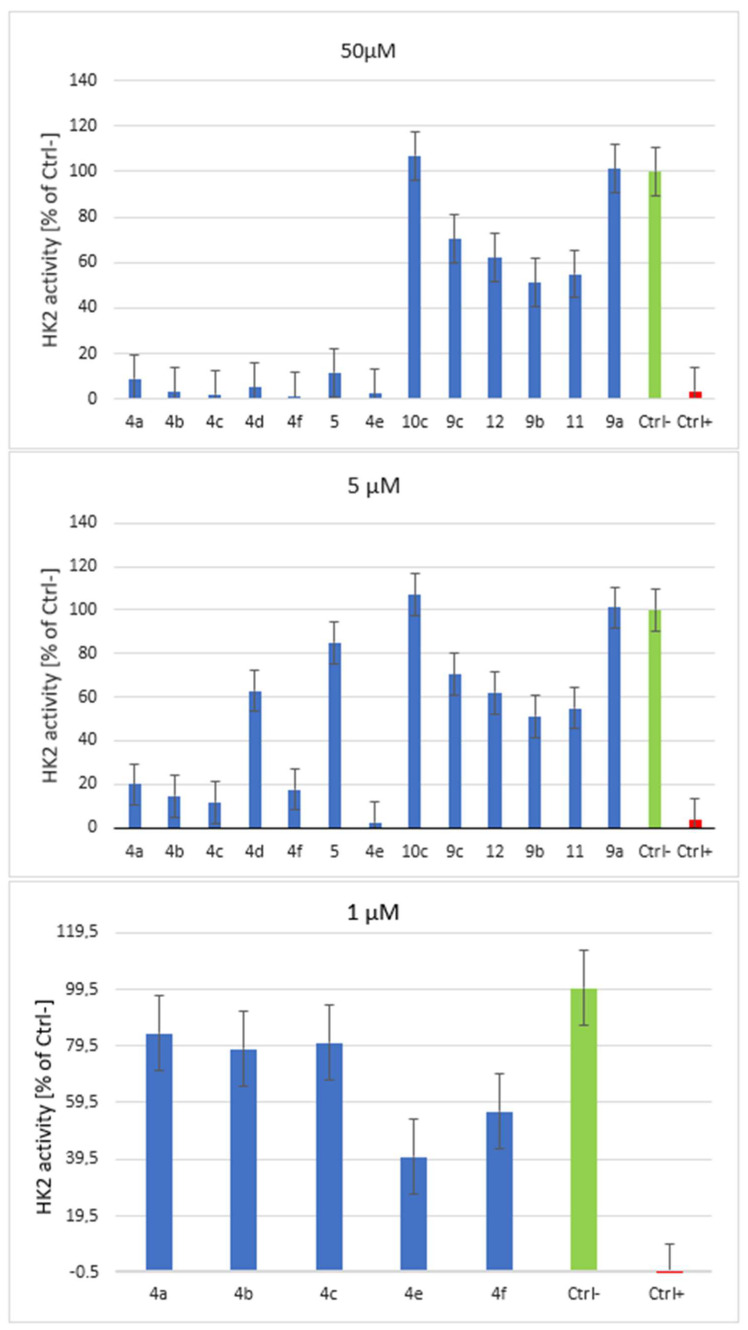
Inhibitory effect of synthesized compounds on HK2 enzyme activity. Bromopyruvic acid was used as a positive HK2 inhibitor control. The relative activity of the negative enzyme control (without inhibitor) was set at 100%. Results are displayed as the mean ± SD from three independent experiments.

Figure 5 and Figure 6 show the results of the HK2 activity inhibition assay of synthesized compounds. Primary measurements were performed at 50 and 5 µM concentrations. We have decided that the concentration of 50 µM is the highest concentration accepted for the investigated compounds as inhibitors. Compounds **4a**, **4b**, **4c**, **4e**, and **4f**, which showed the best inhibition of HK2 activity, were selected for the next assessment at a lower concentration of 1 µM. The data in Figure 5 illustrate the inhibition of HK2-mediated phosphorylation of glucose by the test compounds through changes in absorption detection at λ_max_ 450 nm observed in kinetic mode after 5–45 min.

As shown in Figure 6, at a concentration of 50 µM, most of the compounds show significant HK2 inhibition activity, apart from hydrazides **9**, which show moderate HK2 inhibition. According to the in vitro study, which considered the effect of HK2 inhibition by the tested compounds at a concentration of 5 µM, derivatives **4a**–**4f** exhibited stronger HK2 inhibition activity than the benserazide derivatives **10c**, **11**, and **12**, which have a modified L-amino acid fragment in their structure. The maximum inhibition by benserazide derivatives **10c**, **11**, and **12** was approximately 50%. Hydrazides **9** have no significant effect on HK2 activity at a concentration of 5 µM, which confirms that the presence of three hydroxyl groups is required for the inhibition of HK2 activity.

A comparison of the inhibition results for derivatives **4f** and **5**, for which HK2 inhibition was 92% and 15%, respectively, clearly indicates that the essential feature causing the HK2 inhibitory effect of benitrobenrazide derivatives is the presence of the imine bond (-CH=N-). Referring to studies on the pocket size of the HK2 binding site, it can be assumed that a bulky group, like the anthracenyl group in the structure of the Schiff base **4d**, does not fit efficiently into the active site of HK2. Compared to other Schiff bases with smaller volume substituents **4a**–**c**, compound **4d** did not exhibit inhibitory activity against HK2 at a 5 µM concentration.

The most promising HK2 inhibitors assayed, **4a**, **4b**, **4c**, **4e**, and **4f**, were evaluated against human HK2 at a concentration of 1 µM. The derivatives **4e** and **4f**, which contain a fluorine atom or amino group instead of a nitro group, exhibited the most potent inhibitory effects against HK2 at a concentration of 1 µM, with HK2 inhibition rates of 60% and 54%, respectively. The highest efficacy among the derivatives assessed against HK2 was recorded for Schiff base **4e**, in which the fluorine atom acts as an electron-withdrawing substituent in the para position of the benzene ring.

## 3. Materials and Methods

### 3.1. Chemistry

The ^1^H NMR and ^13^C NMR spectra were recorded using a Varian NMR system 600 spectrometer at 600 MHz in DMSO-d6, with tetramethylsilane (TMS) as the reference standard. NMR chemical shifts are reported in ppm (δ) and coupling constants (J) in Hz. Melting points were measured on a Boethius PHMK apparatus (VEB Analytik Dresden, Dresden, Germany). The progress of the reaction was monitored by thin-layer chromatography (TLC) using Merck TLC silica gel 60 F254 plates and the following developing systems: A: 5% MeOH/CHCl_3_, B: 10% MeOH/CHCl_3_, and C: 20% MeOH/CHCl_3_. Column chromatography was conducted using silica gel 40–60 μm 60A with methanol–chloroform mixtures as eluents. Preparative high-performance liquid chromatography was performed with the LaboACE LC-5060 system (Japan Analytical Industry Co., Ltd., Tokyo, Japan), with an ODS column (JAIGEL-ODS-AP, model SP-12-10, Japan Analytical Industry, Co., Ltd. Tokyo, Japan). The compounds were eluted with a mobile phase of MeOH at a flow rate of 9 mL/min. High-resolution mass spectroscopy (HRMS) was measured on a Waters Corporation Xevo G2 QTOF apparatus (Waters Corporation, Milford, MA, USA) using electrospray ionization (ESI).

#### 3.1.1. General Procedure for the Synthesis of Esters **2e**–**f**

Methyl esters **2a** and **2b** were purchased from Merck. Methyl esters **2c** and **2e**–**2f** were obtained by the well-known method for esterification of acids using thionyl chloride in methanol [37]. To a stirred and cooled (0 °C) solution of the required acid **1c, 1e**–**1f** (20 mmol) in anhydrous methanol (30 mL), thionyl chloride (1.10 equiv.) was added dropwise while stirring. The mixture was warmed up to room temperature and stirred for 24 h. After that, the excess of methanol was removed under diminished pressure and then dried under reduced pressure. In the case of **2f** synthesis, after completion of the esterification reaction, the solution was neutralized by adding saturated aqueous NaHCO_3_ solution until no further gas evolution was observed. Solid **2f** was filtered under reduced pressure.

Synthesis of ethyl anthracene-9-carboxylate **2d**: to SOCl_2_ (7 mL), we added anthracene-9-carboxylic acid (1.80 mmol) and DMF (0.4 mL) as a catalyst. The reaction mixture was stirred at room temperature under argon atmosphere for 4h. The excess of SOCl_2_ was removed under reduced pressure. The residue was washed with toluene. To obtain orange solid acid chloride, EtOH (10 mL) and TEA (1.3 equiv.) were added sequentially. The reaction mixture was stirred at room temperature for 24 h. EtOH was removed under reduced pressure; then, chloroform (10 mL) was added to the residue and the organic layer was washed with water (2 × 8 mL) and dried over anhydrous Na_2_SO_4_. After filtration and evaporation under reduced pressure, the residue was purified on a silica gel packed column using (AcOEt:*n*-hexane 1:1, *v*/*v*) as an eluent, obtaining solid **2d** at a yield of 90%.

#### 3.1.2. General Procedure for the Synthesis of Hydrazides **3a**–**3f**

Hydrazides **3a**–**3f** were synthesized from their corresponding esters **2a**–**2f**, followed by reaction with hydrazine according to the method reported by Khan et al. [10].

Synthesis of hydrazides **3b**–**3c** and **3f**: to a solution of methyl ester **2b**, **c**, or **2f** (20 mmol) in methanol (25 mL), hydrazine monohydrate (80 mmol, 4 equiv.) was added. The reaction mixture was stirred at room temperature for 72 h and then cooled to −20 °C. The formed precipitate was filtered off and dried under reduced pressure. Crystallization of crude solid from an ethanol:water (1:2, *v*/*v*) solution produced white crystals.

Benzohydrazide (**3b**): yield 77%. Mp 114–115 °C. (lit. Mp 110–113 °C [46]). TLC solvent system B; Rf (retention factor): 0.5. ^1^H NMR (600 MHz, DMSO-d6): δ 4.48 (s, N**H_2_**) 7.53–7.46 (m, 2H, C_6_H_5_), 7.49–7.51 (m, 1H, C_6_H_5_), 7.81–7.83 (m, 2H, C_6_H_5_), 9.76 (s, 1N**H**). ^13^C NMR (600 MHz, DMSO-d6): δ 132.08, 133.44, 136.18, 138.46, 171.02. HRMS (ESI-TOF): *m/z* calcd for (C_7_H_8_N_2_O + H^+^): 137.0709; found: 137.0724.

1-naphthohydrazide (**3c**): yield 69%. Mp 156–157 °C (lit. Mp 160–163 °C [38]). TLC system B; Rf: 0.6. ^1^H NMR (600 MHz, DMSO-d6): δ 4.59 (d, J = 3.53 Hz, NH_2_), 7.51–7.58 (m, 4H, C_6_H_5_), 7.96–7.98 (m, 1H, C_6_H_5_), 8.00–8.01 (m, 1H, C_6_H_5_), 8.20–8.22 (m, 1H, C_6_H_5_), 9.68 (s, 1NH) ^13^C NMR 150 MHz, DMSO-d6): 130.16, 130.51, 130.60, 131.40, 131.80, 133.35, 135.02, 135.18, 135.138.30, 138.55, 173.14. HRMS (ESI-TOF): *m/z* calcd for (C_11_H_10_N_2_O + H^+^): 187.0866; found: 187.0610.

4-aminobenzohydrazide (**3f**): yield 60%. Mp 224–226 °C (lit. Mp 225–227 °C [47]). TLC system C; Rf: 0.4. ^1^H NMR (600 MHz, DMSO-d6): δ 4.29 (s, NH_2_), 5.57 (s, C_6_H_5_NH_2_), 6.52 (d, J = 8.72, 2H, C_6_H_5_), 7.54 (d, J = 8.67, 2H, C_6_H_5_), 9.246 (s, 1NH). ^13^C NMR (600 MHz, DMSO-d6): 113.04, 120.40, 128.84, 151.94, 166.88. HRMS (ESI-TOF): *m/z* calcd for (C_7_H_9_N_3_O + H^+^): 152.0818; found: 152.0036.

Synthesis of hydrazides **3a** and **3e**: to a solution of methyl propionate **2a** (5.50 mmol) or methyl 4-fluorobenzoate **2e** (5.50 mmol) in methanol (15 mL), we added hydrazine monohydrate (22 mmol, 4 equiv.). The reaction mixture was stirred at reflux for 24 h and evaporated under diminished pressure. The oily residue **3a** was purified by silica gel column chromatography and eluted with 3% MeOH/CHCl_3_ (*v*/*v*). Product **3e** was crystallized from an ethanol:water (1:2, *v*/*v*) solution.

Propanoic acid hydrazide (**3a**): yield 100%. Mp 36–38 °C (lit. Mp 38–40 °C [48]). TLC system A; Rf: 0.5. ^1^H NMR (600 MHz, DMSO-d6): δ 0.99 (q, J = 7.62, CH_3_), 2.01 (t, J = 7.61, CH_2_), 4.13 (s, NH_2_), 8.90 (s, NH), ^13^C NMR (600 MHz, DMSO-d6): δ 10.37, 27.07, 172.86. HRMS (ESI-TOF): *m/z* calcd for (C_3_H_8_N_2_O + H^+^): 89.0709; found: 89.0736.

4-Fluorobenzhydrazide (**3e**): yield 60%. Mp 164–165 °C. (lit. Mp 160–163 °C [38]). TLC system B; Rf: 0.7. ^1^H NMR (600 MHz, DMSO-d6): δ 4.48 (s, 2NH_2_), 7.24–7.27 (m, 2H, C_6_H_5_), 7.86–7.88 (m, 2H, C_6_H_5_), 9.76 (s, 1NH). ^13^C NMR (600 MHz, DMSO-d6): 115.66 (d, J = 21.75), 129.96 (d, J = 8.98), 163.36, 165.01, 165.31. C_7_H_7_FN_2_O HRMS (ESI-TOF): *m/z* calcd for (C_7_H_7_FN_2_O + H^+^): 155.0615; found: 155.0623.

Synthesis of hydrazide (**3d**): to hydrazine monohydrate (98%, 10 mL), ethyl anthracene-9-carboxylate (**2d**) (1.60 mmol) was added. The reaction mixture was stirred at reflux for 72 h. The residual hydrazine was removed under reduced pressure. A crude solid was purified by flash column chromatography on silica gel, using 100% CHCl_3_ followed by 20% MeOH/CHCl_3_ (*v*/*v*) as an eluent. Additional purification by the high-performance liquid chromatography (HPLC) method using an ODS column with methanol as an eluent was performed.

Anthracene-9-carbohydrazide (**3d**): yield 60%. Td (thermal decomposition temperature) 242 °C. TLC system A; Rf: 0.7. ^1^H NMR (600 MHz, DMSO-d6): δ 4.81 (d, J = 3.10, NH_2_), 7.52–7.57 (m, 4H, C_6_H_5_), 7.98–8.00 (m, 2H, C_6_H_5_), 8.10–8.12 (m, 2H, C_6_H_5_), 8.65 (s, 1H, C_6_H_5_), 9.83 (s, 1NH). ^13^C NMR 600 MHz, DMSO-d6): 125.92, 126.02, 126.76, 127.83, 128.33, 128.77, 131.10, 132.51, 167.96. HRMS (ESI-TOF): *m/z* calcd for (C_15_H_12_N_2_O + H^+^): 237.1028; found: 237.1031.

#### 3.1.3. General Procedure for the Synthesis of Hydrazones (**4a**–**4f**) and Hydrazide (**5**)

Synthesis of hydrazones **4b**–**4c** and **4e**–**4f**: an appropriate hydrazide **3b**,**c**,**e**,**f** (4.40 mmol) and 2,3,4-trihydroxybenzaldehyde (4.40 mmol) were dissolved in anhydrous methanol (15 mL), and the reaction mixture was stirred at room temperature for 24 h. After the completion of the reaction, the formed solid was filtered and purified by recrystallization from EtOH:H_2_O (1:1, *v*/*v*) [13].

(*E*)-*N*′-(2,3,4-trihydroxybenzylidene)benzohydrazide (**4b**): yield 85%. Td 187–189 °C. TLC system B; Rf: 0.4. ^1^H NMR (600 MHz, DMSO-d6): δ 6.41 (d, J = 8.39 Hz, 1H, C_6_H_5_), 6.80 (d, J = 8.48 Hz, 1H, C_6_H_5_), 7.53–7.56 (m, 2H, C_6_H_5_), 7.59–7.62 (m, 1H, C_6_H_5_) 7.93–7.94 (m, 2H, C_6_H_5_)), 8.48 (s, N=CH), 9.46 (s, OH), 11.56 (s, OH), 11.97 (s, NH). ^13^C NMR (600 MHz, DMSO-d6): δ 108.10, 111.28, 121.63, 127.98, 128.96, 132.27, 133.16, 133.38, 147.97, 149.21, 150.63, 162.94. HRMS (ESI-TOF): *m/z* calcd for (C_14_H_12_N_2_O_4_ + H^+^): 273.0870; found: 273.0882.

(*E*)-*N*′-(2,3,4-trihydroxybenzylidene)-1-naphthohydrazide (**4c**): yield 69%. Td 109–110 °C. TLC system C; Rf: 0.4. ^1^H NMR (600 MHz, DMSO-d6): δ 6.38 (d, J = 8.42, 1H, C_6_H_5_), 6.77 (d, J = 8.50, 1H, C_6_H_5_), 7.58–7.61 (m, 3H, C_6_H_5_), 7.76 (dd, J = 1.12, J = 7.03 1H, C_6_H_5_), 8.00–8.02 (m, 1H, C_6_H_5_), 8.09 (d, J = 8.27, 1H, C_6_H_5_), 8.23 (dd, J = 1.51, 8.15 1H, C_6_H_5_), 8.35 (s, N=CH), 8.49 (s, OH), 9.48 (s, OH), 11.47 (s, OH), 12.07 (s, NH) ^13^C NMR (600 MHz, DMSO-d6): δ 108.14, 111.23, 121.60, 125.42, 125.59, 126.44, 126.90, 127.56, 128.81, 130.41, 131.08, 132.77, 133.16, 133.62, 147.99,149.26, 150.51, 164.49. HRMS (ESI-TOF): *m/z* calcd for C_18_H_14_N_2_O_4_ + H^+^): 323.1026; found: 323.1033.

(*E*-4-fluoro-*N*′-(2,3,4-trihydroxybenzylidene)benzohydrazide (**4e**): yield 100%. Td 217–219 °C. TLC system B; Rf: 0.3. ^1^H NMR (600 MHz, DMSO-d6): 6.41(d, J = 8.42, 1H, C_6_H_5_), 6.80 (d, J = 8.49, 1H, C_6_H_5_) 7.38 (t, J = 8.84 2H, C_6_H_5_), 7.99–8.03 (m, 2H, C_6_H_5_), 8.47 (s, N=CH) 11.98 (s, NH) ^13^C NMR (600 MHz, DMSO-d6): δ 108.11, 111.24, 115.95 (d, J_C-F_ = 21.87), 121.66, 130.70 (d, J_C-F_ = 9.12), 133.13, 147.93, 149.21, 150.75, 161.99, 163.80, 165.46. HRMS (ESI-TOF): *m/z* calcd for (C_14_H_11_FN_2_O_4_ + H^+^): 291.0781; found: 291,0875.

(*E*)-4-amino-*N*′-(2,3,4-trihydroxybenzylidene)benzohydrazide (**4f**): yield 72%. Mp > 250 °C. TLC system C; Rf: 0.5. ^1^H NMR (600 MHz, DMSO-d6): δ 5.79 (s, N**H_2_**) 6.38 (d, J = 8.39, 1H, C_6_H_5_), 6.60 (d, J = 8.63, 2H, C_6_H_5_) 6.72 (d, J = 8.48 Hz, 1H, C_6_H_5_), 7.66 (d, J = 8.41, 2H, C_6_H_5_), 8.39 (s, N=CH), 8.44 (s, OH), 9.36 (s, OH), 11.55 (s, OH). 11.78 (s, NH) ^13^C NMR (600 MHz, DMSO-d6): δ 107.93, 111.47, 113.11, 119.41, 121.41, 129.68, 133.13, 147.79, 148.79, 149.01, 152.81, 162.85. HRMS (ESI-TOF): *m/z* calcd for (C_14_H_13_N_3_O_4_ + H^+^): 288.0979; found: 288.0988.

Synthesis of hydrazones **4a**, **4d**: to a solution of anthracene-9-carbohydrazide **3d** (1.14 mmol) or propanoic acid hydrazide **3a** (1.14 mmol) in methanol (15 mL), 2,3,4-trihydroxybenzaldehyde (1.26 mmol, 1.1 equiv.) was added. The reaction mixture was stirred at reflux for 24 h. After consumption of the substrate, the solvent was removed under reduced pressure. The crude solid was crystallized from a solution of ethanol:water (1:1 *v*/*v*).

(*E*)-*N*′-(2,3,4-trihydroxybenzylidene)propionohydrazide (**4a**): yield 89%. Td 184–186 °C. TLC system B; Rf: 0.5. ^1^H NMR (600 MHz, DMSO-d6): δ 1.08 (t, J = 7.57, CH_3_), 2.21 (q, J = 7.55, CH_2_) 6.37 (d, J = 8.41, 1H, C_6_H_5_), 6.73 (d, J = 8.46, 1H, C_6_H_5_), 8.17 (s, N=CH), 8.44 (s, OH), 9.40 (s, OH), 11.07 (s, OH), 11.39 (s, NH). ^13^C NMR (150 MHz, DMSO-d6): 9.41, 26.97, 107.40, 110.66, 120.86, 132.54, 147.20, 148.14, 148.38, 168.70 (HRMS (ESI-TOF): *m/z* calcd for (C_10_H_12_N_2_O_4_ + H^+^): 225.0869; found: 225.0874.

(*E*)-*N*′-(2,3,4-trihydroxybenzylidene)anthracene-9-carbohydrazide (**4d**). yield 50%. Td 167–169 °C. TLC system C; Rf: 0.5. ^1^H NMR (600 MHz, DMSO-d6): δ 6.43 (d, J = 8.37, 1H, C_6_H_5_), 6.82 (d, J = 8.48, 1H, C_6_H_5_) 7.54–7.64 (m, 4H, C_6_H_5_), 8.01–8.02 (m, 2H, C_6_H_5_), 8.16–8.19 (m, 2H, C_6_H_5_), 8.33 (s, N=CH), 8.57 (s, OH), 8.76 (s, 1H, C_6_H_5_), 9.57 (s, OH), 11.43 (s, OH), 12.33 (s, NH). ^13^C NMR 600 MHz, DMSO-d6): δ 108.26, 111.25, 121.61, 125.39, 126.22, 127.32, 127.48, 128.37, 128.99, 129.11, 131.09, 133.22, 148.05, 149.40, 150.59, 164.30. HRMS (ESI-TOF): *m/z* calcd for (C_22_H_16_N_2_O_4_ + H^+^): 373.1188; found: 373.1185.

Synthesis of hydrazide **5**: (*E*)-4-amino-*N*′-(2,3,4-trihydroxybenzylidene)benzohydrazide **4f** (3 mmol), anhydrous methanol (10 mL), and 25% of the weight of hydrazone **4f** 20% Pd(OH)_2_/C were added to a reaction vessel. The vessel was placed in a Parr hydrogenator and the reaction mixture was treated with hydrogen at 2.2 bar at room temperature for 6 h. The catalyst was filtered from the solution and the reaction mixture was concentrated under reduced pressure. The crude solid was crystallized from an ethanol solution.

4-amino-*N*′-(2,3,4-trihydroxybenzyl)benzohydrazide (**5**): yield 40%. Td 188–190 °C. TLC system B; Rf: 0.2.^1^H NMR (600 MHz, DMSO-d6): δ 3.80 (d, J = 3.80, N-CH_2_), 5.18 (d, J = 5.07, NH-CH_2_), 5.63 (s, NH_2_) 6.20 (d, J = 8.11, 1H, C_6_H_5_), 6.42 (d, J = 8.18, 1H, C_6_H_5_) 6.53 (d, J = 8.54, 2H, C_6_H_5_), 7.54 (d, J = 8.50, 2H, C_6_H_5_) 8.12 (s, OH), 8.74 (s, OH), 9.20 (s, OH), 9.74 (d, J = 3.27, NH) ^13^C NMR (600 MHz, DMSO-d6): δ 52.50, 106.56, 113.00, 115.65, 119.58, 119.85, 129.07, 133.56, 145.81, 145.98, 152.25, 166.39. HRMS (ESI-TOF): *m/z* calcd for (C_14_H_15_N_3_O_4_ + Na^+^); 312.0955; found: 312.0955.

#### 3.1.4. General Procedure for the Synthesis of Amino Acid Methyl Ester Hydrochloride **7a**–**7c**

Methyl esters **7a**–**7c** were obtained by the well-known amino acid esterification method, which uses thionyl chloride in methanol [25]. To a stirred and cooled (0 °C) solution of required L-amino acid **6** (30 mmol) in anhydrous methanol (40 mL), SOCl_2_ (1.10 equiv.) was added dropwise. The reaction mixture was warmed up to room temperature and stirred for 24 h. After completion of the reaction, excess methanol was removed and dried under reduced pressure.

#### 3.1.5. General Procedure for the Synthesis of N-Benzyloxycarbonyl-L-amino Acid Methyl Esters **8a**–**8c**

An amino group of derivatives **7a**–**7c** was protected using benzyl chloroformate [25]. To a solution of amino acid methyl ester hydrochloride salt **7a**–**7c** (25 mmol) in dichloromethane (30 mL), triethylamine (2.5 equiv.) was added. After 10 min, benzyl chloroformate (1.2 equiv.) was added dropwise to the reaction mixture at 0 °C. The reaction mixture was stirred at room temperature for 24 h; then, water was added to solubilize all salts. The organic layer was washed with water (2 × 30 mL) and dried over anhydrous Na_2_SO_4_. After filtration, the organic layer was concentrated under reduced pressure. The residue was purified on a silica gel column using a mixture of AcOEt:*n*-hexane, 1:1 *v*/*v*, obtaining colorless oil **8a**–**8c**.

#### 3.1.6. General Procedure for the Synthesis of N-Benzyloxycarbonyl-L-amino Acid Hydrazides **9a**–**9c**

To a solution of *N*-Cbz-amino acid methyl esters **8a**–**8c** (16.5 mmol) in anhydrous methanol (30 mL), hydrazine hydrate (98% 4.0 equiv.) was added. The reaction mixture was stirred for 24 h at room temperature. The insoluble product formed was filtered off and was recrystallized from a solution of ethanol: H_2_O, 1:1 *v*/*v* [25].

*N*-benzyloxycarbonyl-L-glycine hydrazide (**9a**): yield 97%. Mp 114-115 °C (lit. Mp 112–114 °C [49]). TLC system B; Rf: 0.8. ^1^H NMR (600 MHz, DMSO-d6): δ 3.55 (d, J = 6.16, CH_2_), 4.17(s, NH_2_), 5.00 (s, CH_2_C_6_H_5_), 7.28–7.39 (m, 5H, C_6_H_5_), 9.02 (s, 1NH), ^13^C NMR (600 MHz, DMSO-d6): δ 42.65, 65.91, 128.13, 128.23, 128.77, 137.44, 156.86, 168.93. HRMS (ESI-TOF): *m/z* calcd for (C_10_H_13_O_3_N_3_ + H^+^): 224.1030; found: 224.1046.

*N*-benzyloxycarbonyl-L-tyrosine hydrazide (**9b**): yield 36%. Mp 218–219 °C. (lit. Mp 219–221 °C [50]). TLC system B; Rf: 0.8. ^1^H NMR (600 MHz, DMSO-d6): δ 2.62–2.66 (m, HCH), 2.77–2.81 (m, HCH), 4.08–4.12 (m, CH), 4.21 (s, NH_2_) 4.91–4.96 (m, CH_2_C_6_H_5_), 5.04 (s, OH), 6.65 (d, J = 8.36, 2H, C_6_H_5_), 7.04 (d, J = 8.30, 2H, C_6_H_5_), 7.24–7.35 (m, 3H, C_6_H_5_), 7.44 (d, J = 8.71, 2H, C_6_H_5_), 9.17 (s, 1NH), 9.18 (s, NHCbz) ^13^C NMR (600 MHz, DMSO-d6): δ 37.48, 55.72, 65.57, 115.30, 127.86, 128.07, 128.49, 128.70, 128.76, 130.52, 137.52, 156.13, 156.19, 171.29. (ESI-TOF): *m/z* calcd for (C_11_H_15_N_3_O_3_S + H^+^): 330.1454; found: 330.1441.

*N*-benzyloxycarbonyl-L-cysteine hydrazide (**9c**): yield 67%. Mp 138–139 °C (lit. Mp 141–143 °C [51]). TLC system B; Rf: 0.7. ^1^H NMR (600 MHz, DMSO-d6): δ 1.23 (s, SH), 2.84–2.88 (m, CH), 3.03–3.06 (m, CH), 4.26 (s, NH_2_), 5.00–5.04 (m, CH_2_C_6_H_5_), 7.31–7.36 (m, 4H, C_6_H_5_), 7.56 (d, J = 8.53, 1H, C_6_H_5_) 9.30 (s, NH, NHCbz) ^13^C NMR (600 MHz, DMSO-d6): δ 53.00, 56.47, 66.00, 128.16, 128.23, 128.75, 137.33, 156.24, 169.60. HRMS (ESI-TOF): *m/z* calcd. for (C_7_H_8_ON_2_ − H^−^): 268.0761; found: 268.0763.

#### 3.1.7. General Procedure for the Synthesis of Hydrazones of N-Benzyloxycarbonyl-amino Acids **10a**–**10c**

Synthesis of (*E*)-benzyl(2-oxo-2-(2-(2,3,4-trihydroxybenzylidene)hydrazinyl)ethyl) carbamate: 2,3,4-trihydroxybenzaldehyd (11.7 mmol; 1.20 equiv.) was added to a stirring solution of *N*-Cbz-L-glycine hydrazide **9a** (9.8 mmol) in methanol (25 mL). The mixture solution was stirred at room temperature for 24 h. After completion of the reaction, the obtained solid was filtered off. The product was obtained as a solid and purified by recrystallization from the solution of methanol: H_2_O (1:1 *v*/*v*).

Synthesis of (*E*)-benzyl(3-(4-hydroxyphenyl)-1-oxo-1-(2-(2,3,4-trihydroxybenzylidene)- hydrazinyl)propan-2-yl)carbamate **10b**: 2,3,4-trihydroxybenzaldehyd (1.20 mmol; 1.20 equiv.) was added to a stirring solution of *N*-benzyloxycarbonyl-L-tyrosine hydrazide **9b** (1.00 mmol) in THF (10 mL). The resulting solution was stirred at reflux for 72 h in an inert atmosphere of argon. The reaction mixture was concentrated under reduced pressure. The residue was crystallized from a 1:1 (*v*/*v*) solution of methanol:H_2_O at a 55% yield.

Synthesis of (*E*)-benzyl(3-mercapto-1-oxo-1-(2-(2,3,4-trihydroxybenzylidene)hydrazinyl)propan-2-yl)carbamate **10c**: 2,3,4-trihydroxybenzaldehyd (16.92 mmol; 1.20 equiv.) was added to a stirring solution of *N*-Cbz-L-cysteine hydrazide **9c** (14.10 mmol) in methanol (30 mL). The mixture solution was stirred at room temperature for 24 h and concentrated under reduced pressure. The residue was crystallized from an ethanol:H_2_O, 1:1 *v*/*v* solution.

(*E*)-benzyl(2-oxo-2-(2-(2,3,4-trihydroxybenzylidene)hydrazinyl)ethyl)carbamate (**10a**): yield 97%. Mp 213–215 °C (Mp 211–212 °C [52]). TLC system B; Rf: 0.3. ^1^H NMR (600 MHz, DMSO-d6): δ 3.75 (d, J = 4.41, CH_2_), 5.06 (s, CH_2_C_6_H_5_), 6.38 (d, J = 7.92, 1H, C_6_H_5_) 6.77 (d, J = 7.87, 1H, C_6_H_5_), 7.32–7.37 (m, 4H, C_6_H_5_), 7.60 (s, 1H, C_6_H_5_), 8.25 (s, N=CH), 8.47 (s, OH) 9.45, (s, OH), 9.52 (d, J = 15.50, NHCbz), 11.32 (s, OH), 11.54 (s, NH) ^13^C NMR (600 MHz, DMSO-d6): δ 43.09 66.03, 108.04, 111.16, 121.48, 128.11, 128.19, 128.26, 128.79, 133.11, 137.41, 147.80, 149.12, 149.62, 157.00, 165.65. HRMS (ESI-TOF): *m/z* calcd for (C_17_H_17_N_3_O_6_ + H^+^): 360.1190; found: 360.1197.

(*E*)-benzyl(3-(4-hydroxyphenyl)-1-oxo-1-(2-(2,3,4-trihydroxybenzylidene)hydrazinyl)propan-2-yl)carbamate (**10b**): yield 55%. Mp 106–108 °C. TLC system B; Rf: 0.3. ^1^H NMR (600 MHz, DMSO-d6): δ 2.72–2.746 (m, HCH), 2.88–2.91 (m, HCH), 4.20–4.21 (m, CH), 4.94–5.00 (m, CH_2_C_6_H_5_), 6.66 (d, J = 8.16, 1H, C_6_H_5_), 6.38 (d, J = 8.40, 1H, C_6_H_5_), 6.77 (d, J = 8.47, 1H, C_6_H_5_), 7.08 (d, J = 8.00, 2H, C_6_H_5_), 8.24 (s, N=CH), 8.46 (s, OH), 9.21 (s, NHCbz) 9.46 (s, OH), 11.27 (s, OH) ^13^C NMR (600 MHz, DMSO-d6): δ 36.96, 56.11, 65.76, 108.05, 111.18, 115.37, 121.45, 127.87, 127.94, 128.15, 128.70 128.73, 130.56, 133.10, 137.40, 147.81, 149.14, 149.86, 156.29, 168.02. HRMS (ESI-TOF): *m/z* calcd for (C_24_H_23_N_3_O_7_ + H^+^); 466.1609; found: 466.1610.

(*E*)-benzyl(3-mercapto-1-oxo-1-(2-(2,3,4-trihydroxybenzylidene)hydrazinyl)propan-2-yl)carbamate (**10c**): yield 49%. Mp 134–135 °C. TLC system B; Rf: 0.2. ^1^H NMR (600 MHz, DMSO-d6): δ 1.24 (s, SH), 2.96–2.99 (m, HCH), 3.18-3.21 (m, HCH), 4.39 (dd, J = 8.54, 14.07 CH), 5.05 (s, CH_2_C_6_H_5_), 6.37 (d, J = 8.41, 1H, C_6_H_5_), 6.75 (d, J = 8.41, 1H, C_6_H_5_), 7.30–7.37 (m, 4H, C_6_H_5_), 7.84 (d, J = 8.21, 1H, C_6_H_5_), 8.31 (s, N=CH), 8.48 (s, OH), 9.14 (s, NHCbz), 9.48 (s, OH), 11.25 (s, OH), 11.78 (s, NH). ^13^C NMR (600 MHz, DMSO-d6): δ 53.50, 65.97, 66.18, 108.10, 111.13, 121.56, 128.21, 128.29, 128.78, 133.10, 137.21, 147.88, 149.26, 150.40, 156.40, 166.49. HRMS (ESI-TOF): *m/z* calcd for (C_18_H_19_N_3_O_6_S − H^−^): 404.0921; found: 404.0916.

#### 3.1.8. General Procedure for the Synthesis of Substituted Amino Acid Hydrazide **11** and Substituted Amino Acid Hydrazone **12**

Synthesis of 2-amino-*N*′-(2,3,4-trihydroxybenzyl)acetohydrazide **11**: ammonium formate (1 mmol, 1 equiv.), 40% (*w*/*v*) hydrazone **10a**, and 10% PdOH_2_ (145 mg) were added to a stirring solution of *N*-(*N*-Cbz-L-glycine)-2,3,4-trihydroxybenzaldehyde hydrazone **10a** (1 mmol) in methanol (10 mL). The reaction mixture was stirred at 50 °C under an argon atmosphere for 12 h then was cooled down to room temperature, filtered to remove the catalyst, and concentrated under reduced pressure. The residue was purified by crystallization from diethyl ether and the pure powder was lyophilized. The hygroscopic powder was obtained in 48% yield.

Synthesis of (*E*)-2-amino-3-(4-hydroxyphenyl)-*N*′-(2,3,4-trihydroxybenzylidene)propanehydrazide **12**: hydrazone **10b** (0.5 mmol), anhydrous methanol (30 mL), and 25% (*w*/*v*) hydrazone **10b** were added to a mixture of 10% Pd/C and Pd(OH)_2_. The reaction vessel was placed in a Parr shaker hydrogenator at pressures of up to 2.5 bar at room temperature for 6 h. The solid catalyst was filtered off and the reaction solution was concentrated under reduced pressure. The product was purified by reverse-phase preparative HPLC on an ODS column using 70% MeOH:H_2_O (*v*/*v*).

2-Amino-*N*′-(2,3,4-trihydroxybenzyl)acetohydrazide (**11**): yield 48%. Hygroscopic powder. TLC system B; Rf: 0.1. ^1^H NMR (600 MHz, DMSO-d6): δ 1.99 (s, NH_2_), 3.06 (s, CH_2_), 3.49 (s, NH-CH_2_), 3.60 (s, NH-CH_2_), 6.12 (d, J = 8.06, 1H, C_6_H_5_), 6.28 (d, J = 8.09, 1H, C_6_H_5_), 8.86 (s, NH). ^13^C NMR (600 MHz, DMSO-d6): δ 43.17, 44.11, 106.75, 115.31, 119.81, 133.78, 145.00, 145.16, 172.52. HRMS (ESI-TOF): *m/z* calcd for (C_9_H_13_N_3_O_4_ + H^+^) 228.0978; found: 228.0331.

(*E*)-2-Amino-3-(4-hydroxyphenyl)-*N*′-(2,3,4-trihydroxybenzylidene)propanehydrazide (**12**): yield 20%. Td 176–178 °C TLC system C; Rf: 0.2. ^1^H NMR (600 MHz, DMSO-d6): δ 2.58–2.61 (m, HCH), 2.76–2.79 (m, HCH), 3.65–3.69 (m, CH), 4.41 (d, J = 7.43, NH), 4.51 (d, J = 7.37, NH), 6.18–6.21 (m, 1H, C_6_H_5_), 6.36–6.40 (m, 1H, C_6_H_5_), 6.62–6.68 (m, 2H, C_6_H_5_), 6.91–7.07 (m, 2H, C_6_H_5_), 8.04 (s, N=CH), 8.31 (s, NH) ^13^C NMR (600 MHz, DMSO-d6): δ 29.46, 55.21, 106.66, 115.39, 119.68, 128.29, 128.45, 130.57, 130.65, 133.46, 145.63, 146.01, 156.26, 172.87 HRMS (ESI-TOF): *m/z* calcd for (C_16_H_17_N_3_O_5_ + H^+^): 332.1241; found: 332.1253.

^1^H and ^13^C NMR spectra of synthesized compounds are presenting in Supplementary Materials.

### 3.2. Computational Methods

Calculations were performed by the Orca 4.2.1 package on the DFT level (B3LYP/def2-SVP) [53]. The accuracy of the optimization process was determined using the Hessian eigenvalue analysis. All the calculated Hessian eigenvalues were positive for the compounds evaluated. In the case of calculations in a solvent environment, the CPCM continuous solvation model for DMSO was used.

### 3.3. Hexokinase Activity Assay

For studying the potential of HK2 inhibitors, a commercially available assay test (ab211114) Hexokinase II Inhibitor Screening (colorimetric) was used. The in vitro hexokinase activity assay was conducted according to the manufacturer’s instructions. Briefly, the enzyme and substrate solution were prepared. Next, the enzyme solution was added to the wells containing sample compounds and incubated for 5 min at 25 °C. Then, the substrate solution was added to the wells and the absorbance was measured at 450 nm every 5 min for 45 min using the Thermo Scientific™ Varioskan™ LUX multimode microplate reader (ThermoFisher Inc.Waltham, MA, USA). The test compounds were dissolved in DMSO at 50 µM, 5 µM, and 1 µM concentrations, with the final concentration of solvent not exceeding 1% by volume.

## 4. Conclusions

In our research, we synthesized benitrobenrazide and benserazide analogues. We identified that some of these compounds, namely compounds **4e** and **4f**, represent a promising class of HK2 inhibitors, inhibiting HK2 at a concentration of 5 µM by 98% and 82%, respectively. At the lower concentration of 1 µM, **4e** and **4f** inhibited HK2 by 60% and 54%, respectively. We have confirmed that the presence of a bulky anthracenyl group in **4d** has no significant effect on HK2 enzymatic activity. The exchanging of serine by glycine or threonine in benserazide analogues has a minor effect on their inhibition activity against HK2. Compounds **11** and **12** reduce the enzymatic activity of HK2 by approx. 40% in comparison with the negative control. The presented findings suggest that the imine scaffold -CH=N-, in the structure of the potent HK2 inhibitors, helps to enhance and regulate their biological activities. The -CH=N- core is responsible for the possible binding of various groups with nucleophilic and electrophilic properties and, thus, can interact with targeted enzymes and inhibit their enzymatic activity.

## Data Availability

Data are contained within the article and supplementary materials.

## Supplementary Materials

The following supporting information can be downloaded at: https://www.mdpi.com/article/10.3390/molecules29030629/s1, Figure S1: Hydrogen bonding interaction in compound **4a**; ^1^H and ^13^C NMR spectra of synthesized compounds. Figures S2–S27: ^1^H and ^13^C NMR spectrum of synthesized compounds.
