# Supplementary material for: Synthesis and Primary Activity Assay of Novel Benitrobenrazide and Benserazide Derivatives
Source: Molecules. 2024 Jan 29;29(3):629. doi: 10.3390/molecules29030629 (PMC10856005; doi:10.3390/molecules29030629)
Supplement: Supplementary file 1 [file molecules-29-00629-s001.zip › molecules-2831296-supplementary.pdf]

Synthesis and Primary Activity Assay of Novel Benitrobenrazide and Benserazide Derivatives

Karolina Juszczak, Wojciech Szczepankiewicz and Krzysztof Walczak *

Department of Organic Chemistry, Bioorganic Chemistry and Biotechnology, Faculty of Chemistry,
Silesian University of Technology, 44-100 Gliwice, Poland; karolina.juszczak@polsl.pl (K.J.); wojciech.szczepankiewicz@polsl.pl (W.S.)
*   Correspondence: krzysztof.walczak@polsl.pl

Supporting Information

1. Hydrogen bonding interaction in compound **4a** **S1**
2. NMR spectra of compounds evaluated for inhibition of HK2. ....**S2–S27**

Figure S1: Hydrogen bonding interaction in compound **4a**

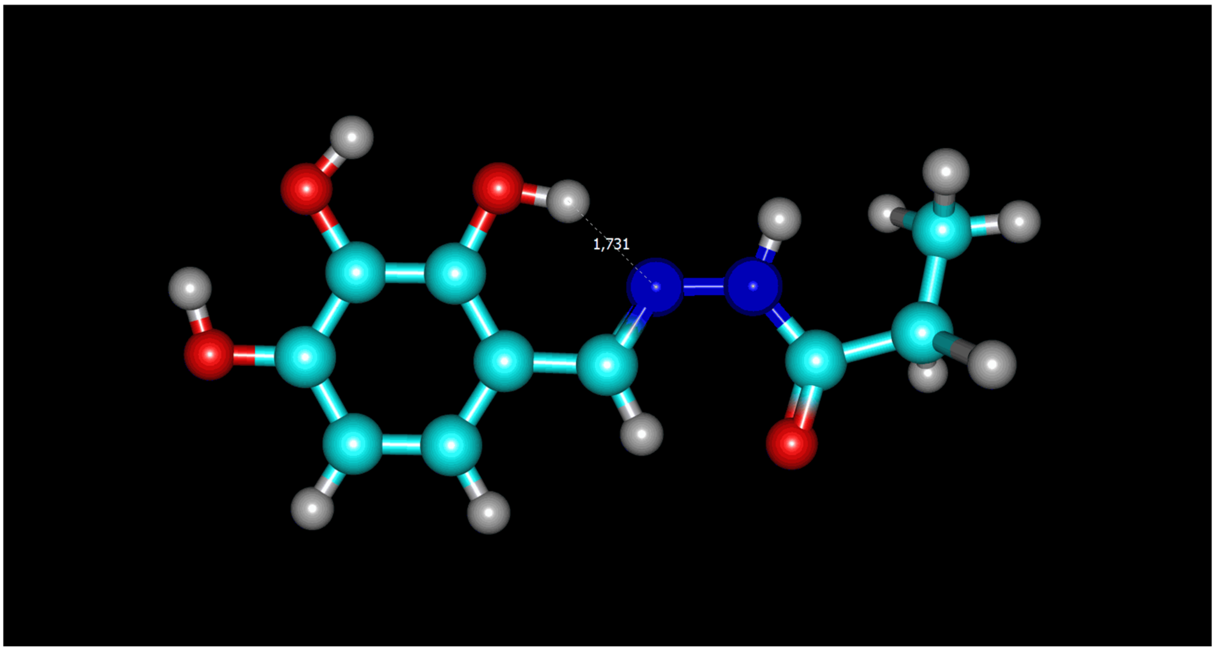

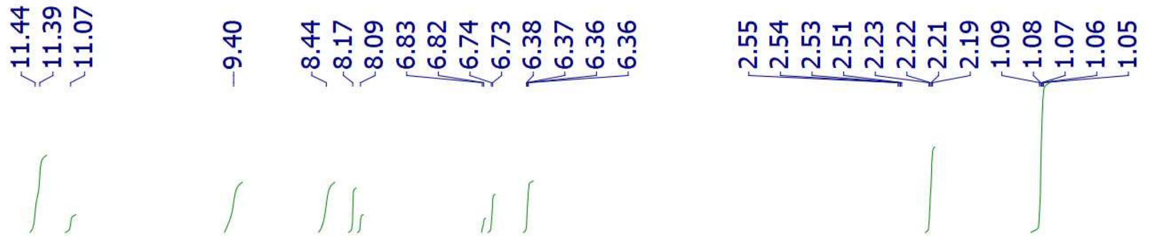

Figure S2: ^{1}H NMR spectrum of **4a** (DMSO, 600 MHz)

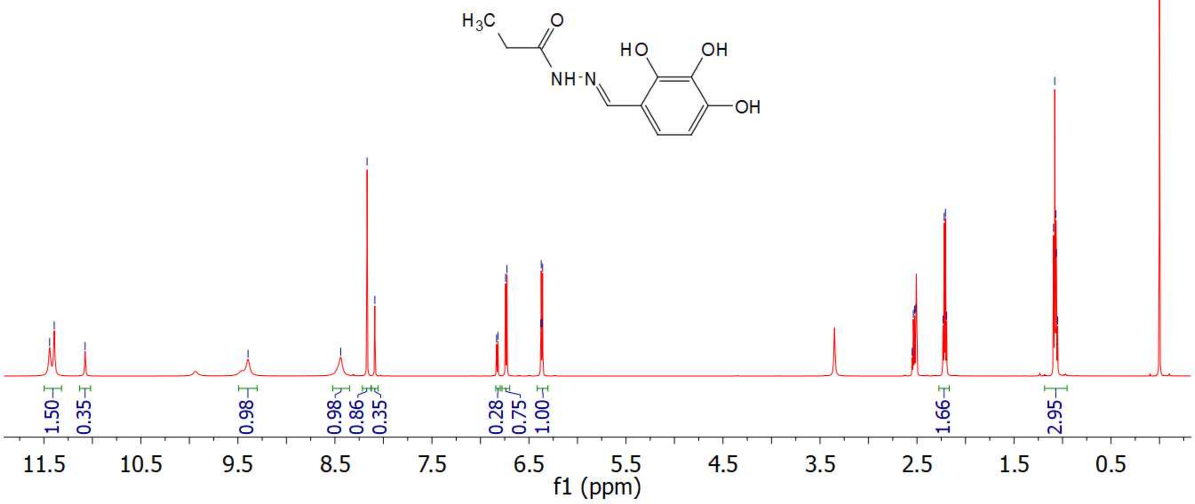

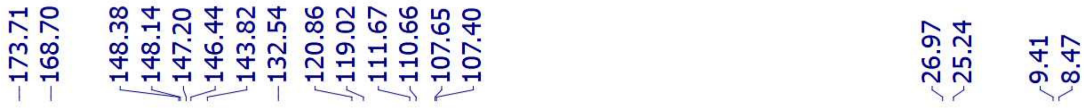

Figure S3: ^{13}C NMR spectrum of **4a** (DMSO, 600 MHz)

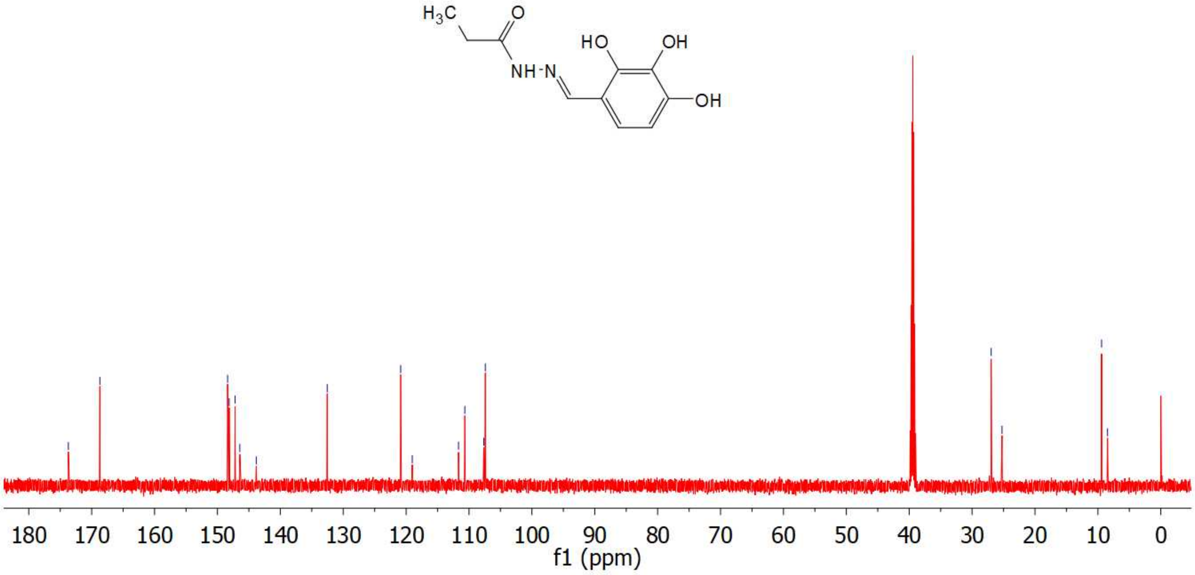

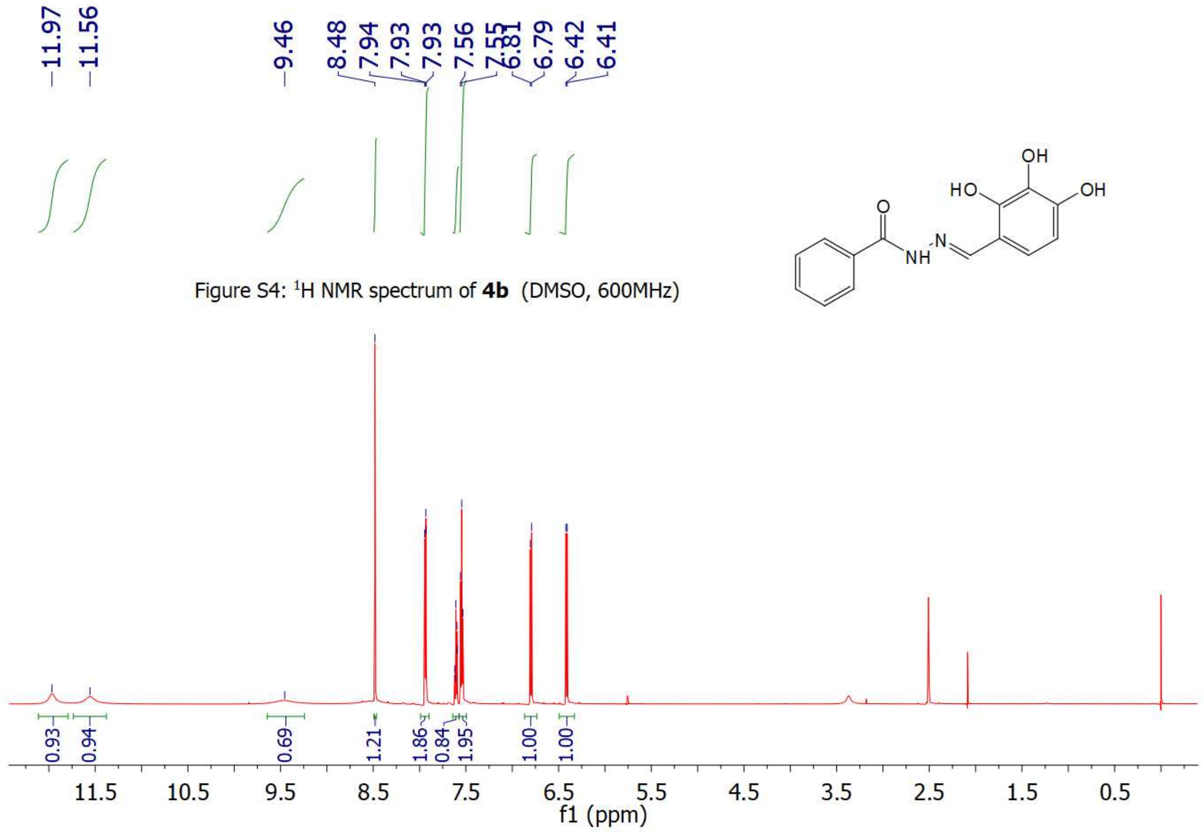

Figure S4: ^{1}H NMR spectrum of **4b** (DMSO, 600MHz)

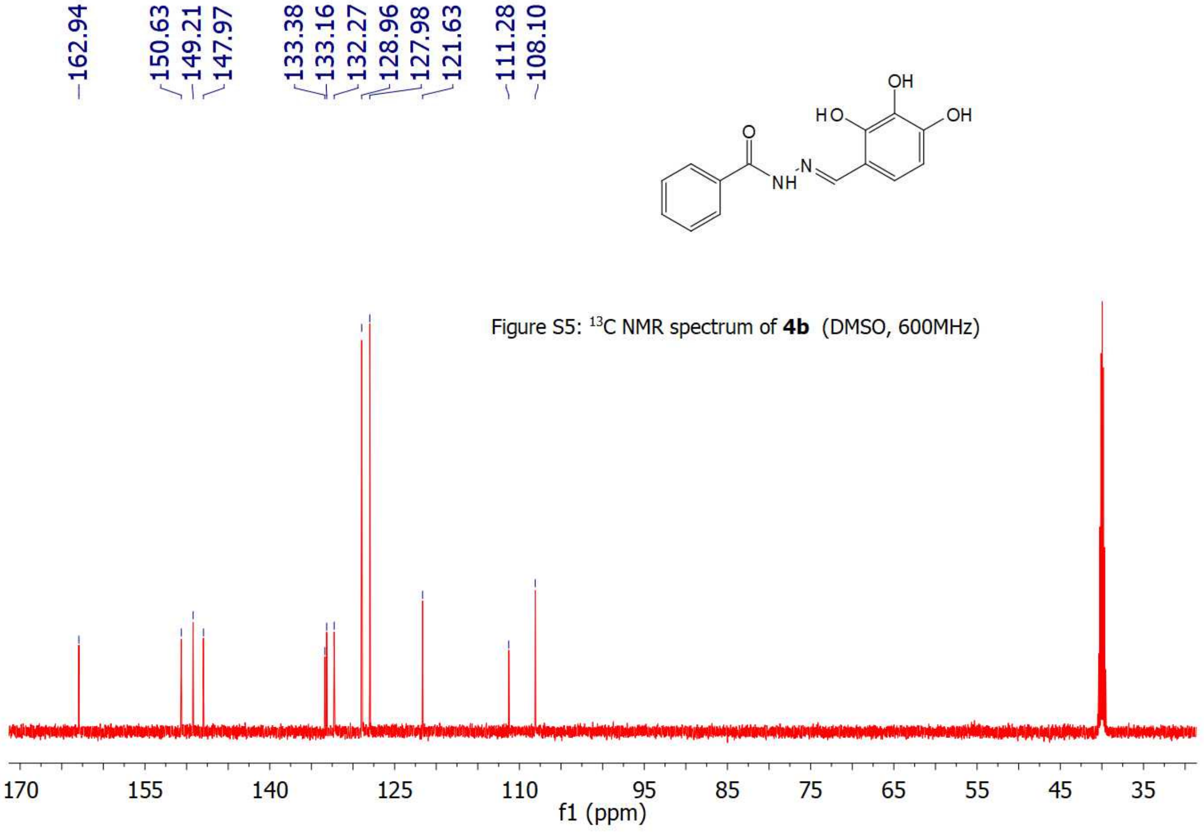

Figure S5: ^{13}C NMR spectrum of **4b** (DMSO, 600MHz)

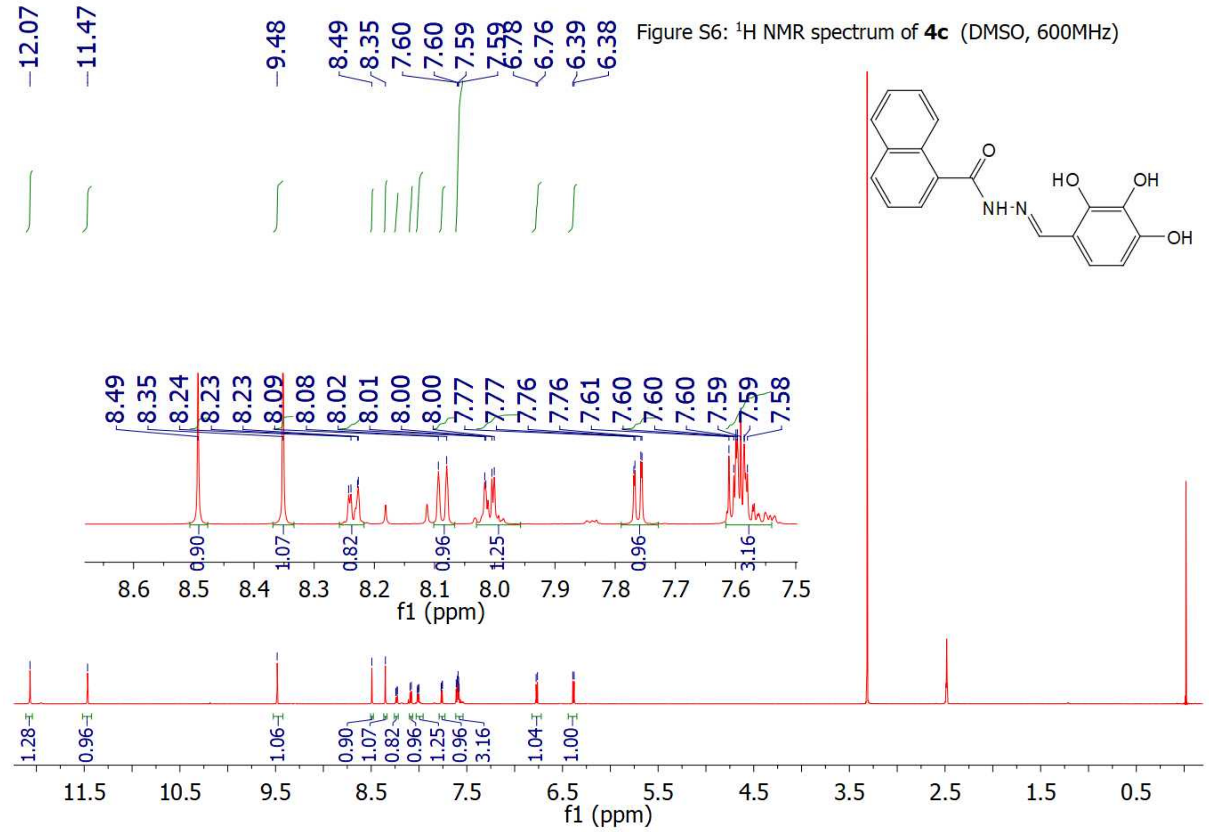

Figure S6: ^{1}H NMR spectrum of **4c** (DMSO, 600MHz)

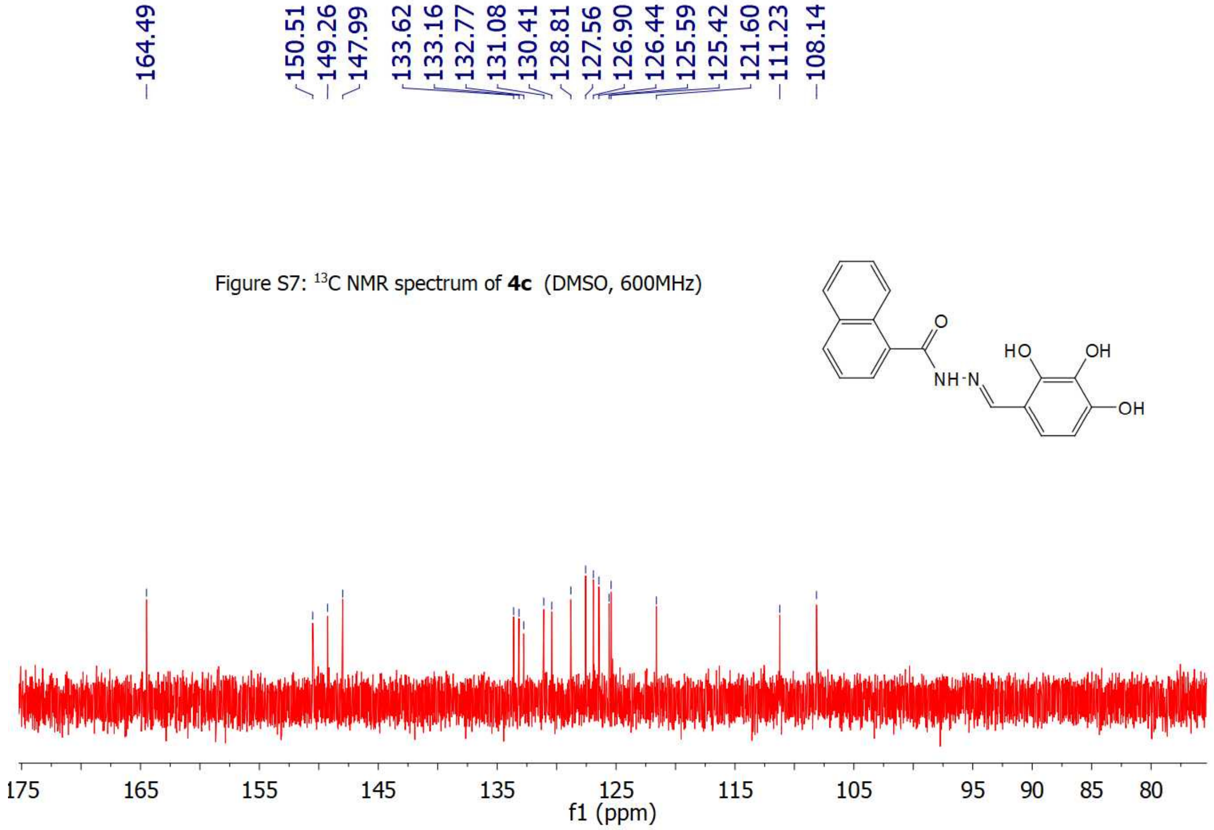

Figure S7: ^{13}C NMR spectrum of **4c** (DMSO, 600MHz)

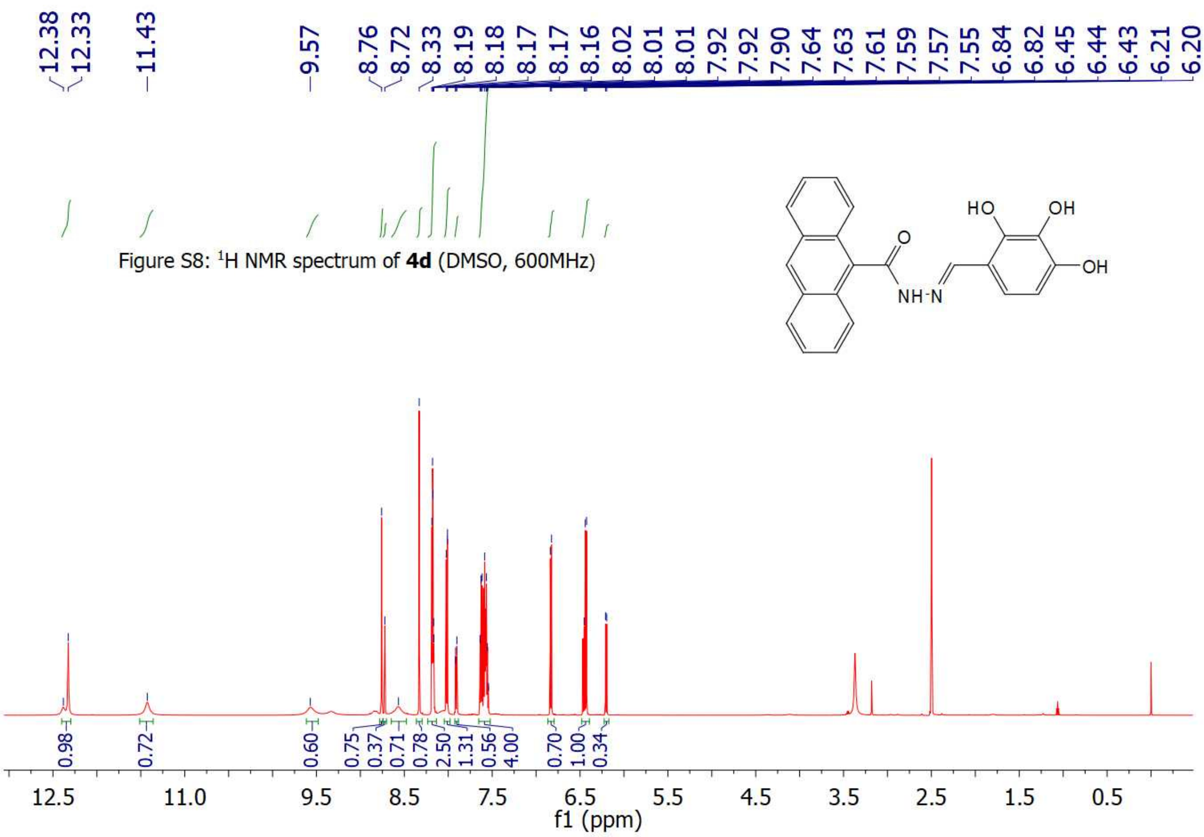

Figure S8: ^{1}H NMR spectrum of **4d** (DMSO, 600MHz)

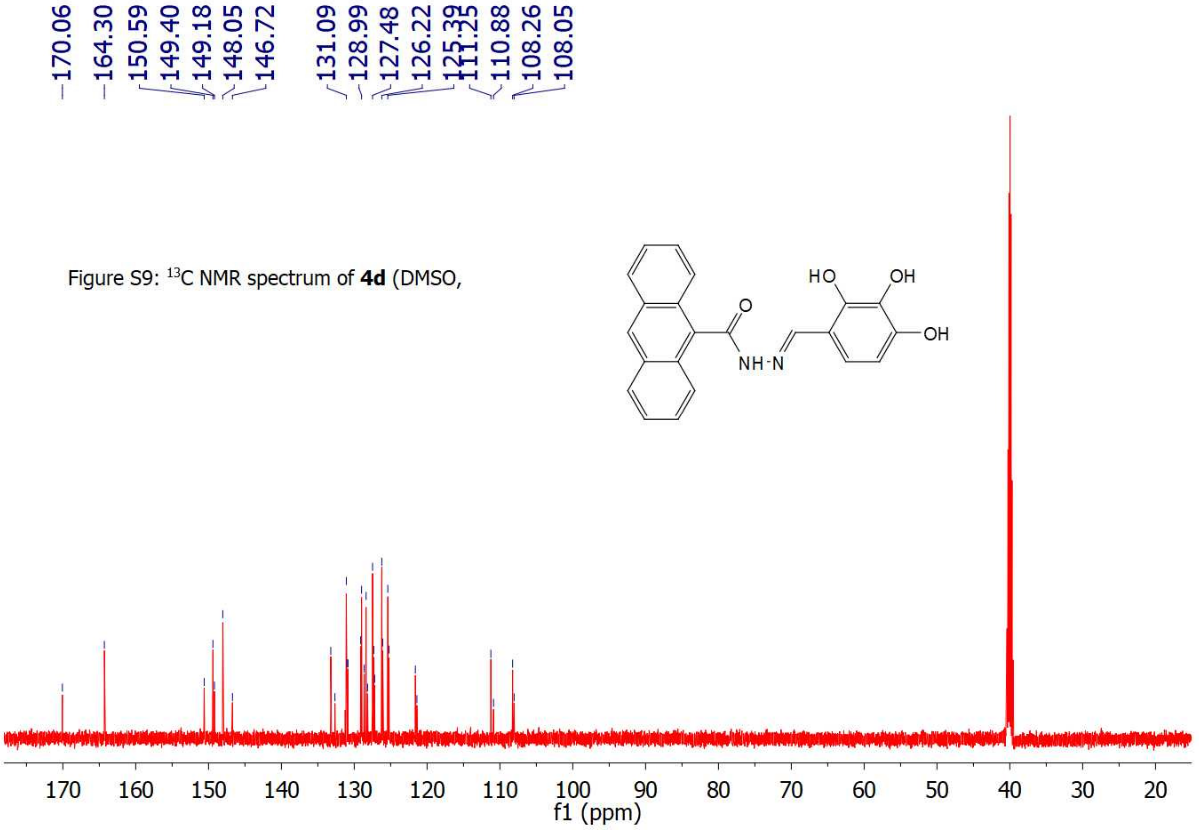

Figure S9: ^{13}C NMR spectrum of **4d** (DMSO,

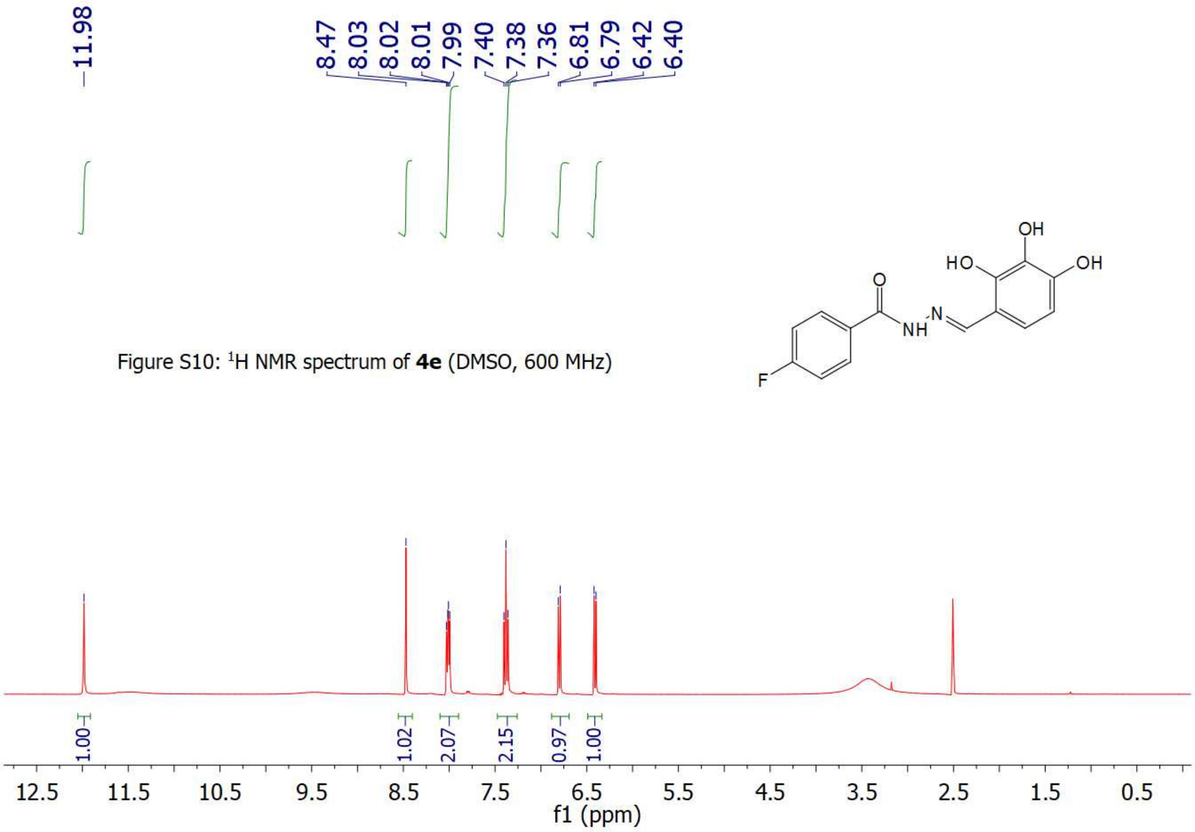

Figure S10: ^{1}H NMR spectrum of **4e** (DMSO, 600 MHz)

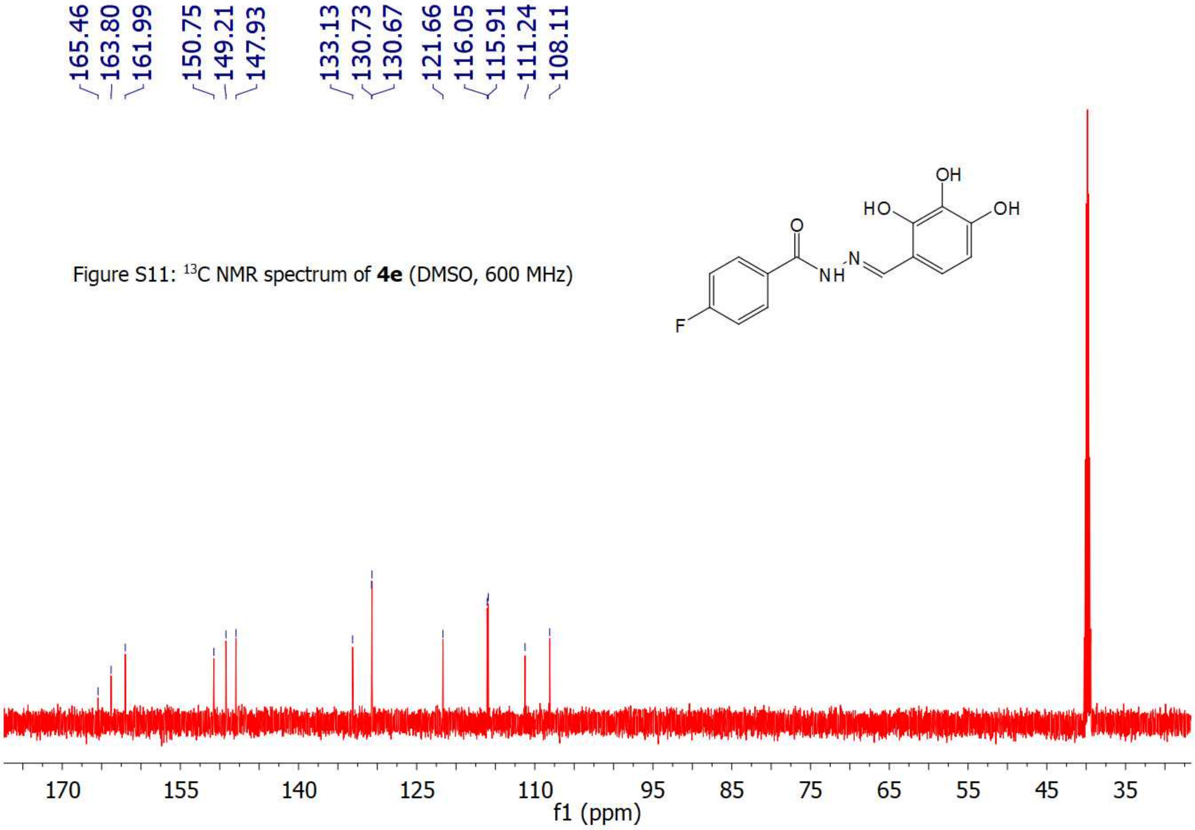

Figure S11: ^{13}C NMR spectrum of **4e** (DMSO, 600 MHz)

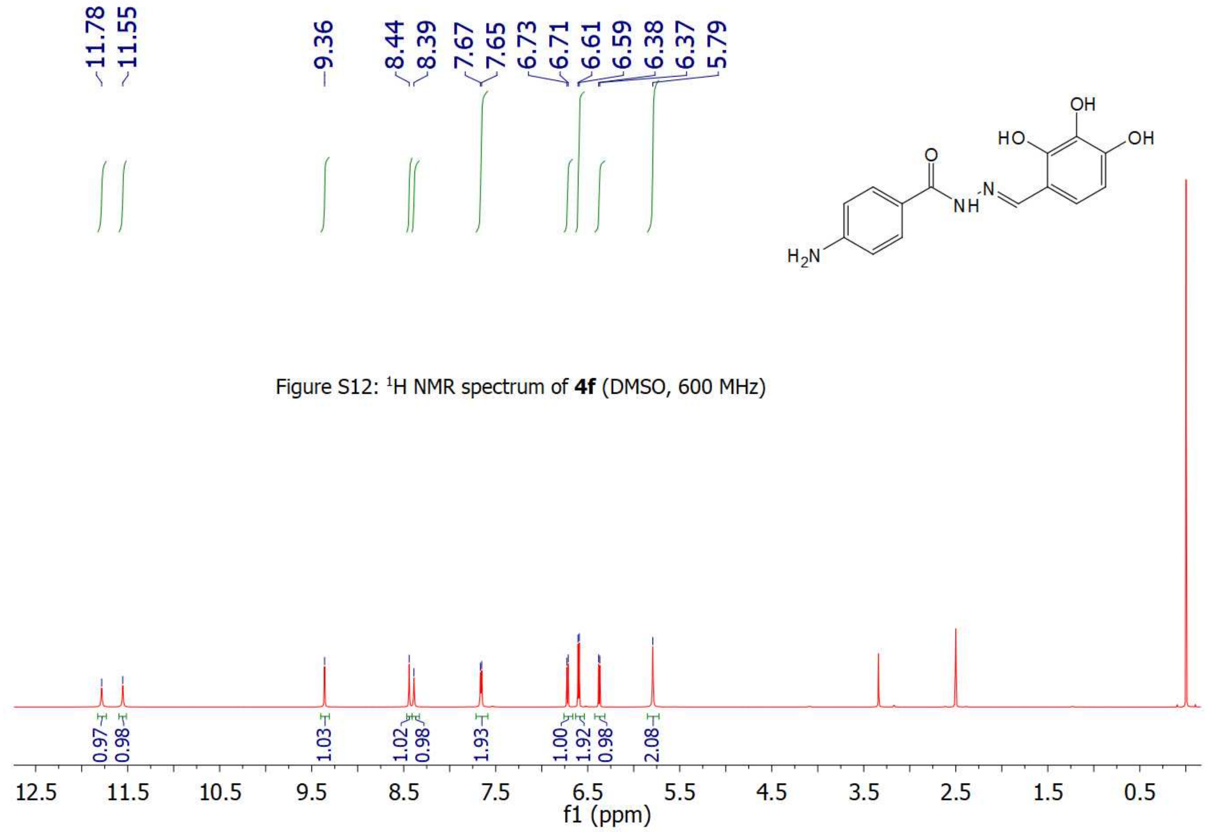

Figure S12: ^{1}H NMR spectrum of **4f** (DMSO, 600 MHz)

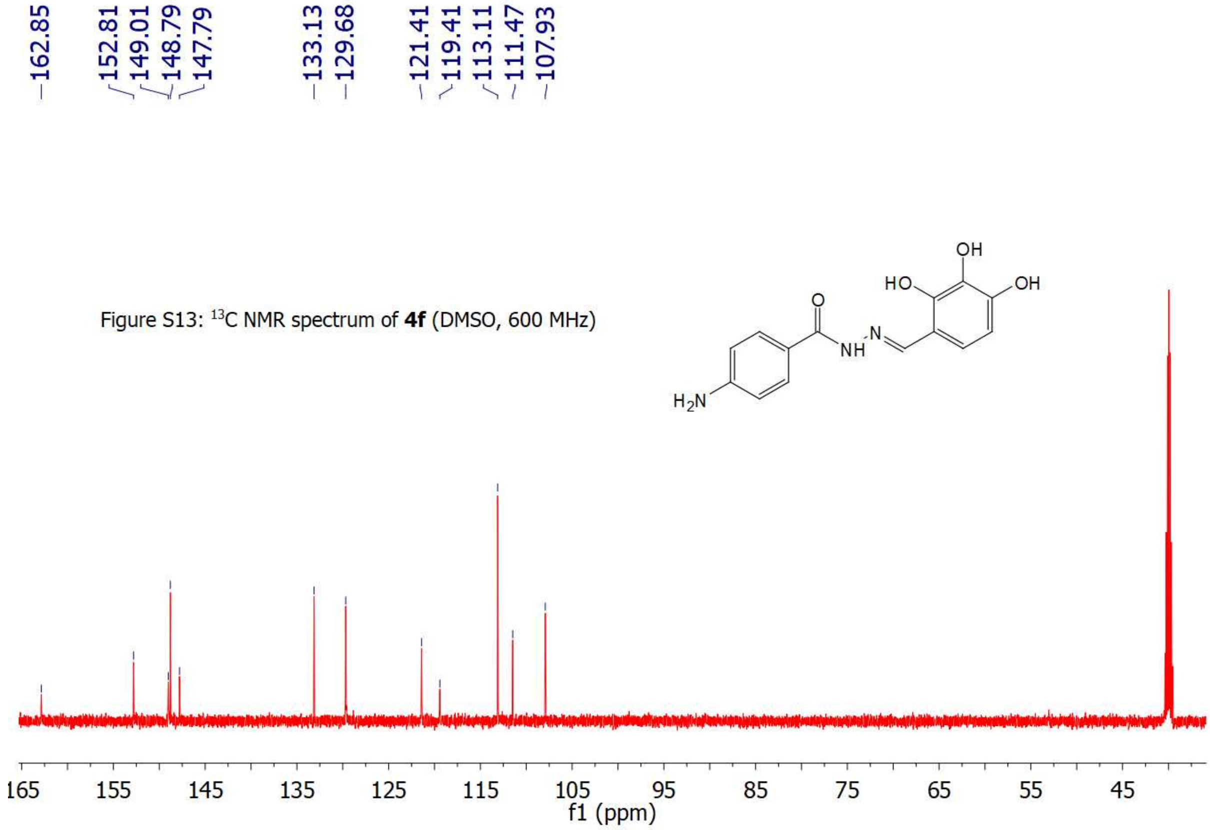

Figure S13: ^{13}C NMR spectrum of **4f** (DMSO, 600 MHz)

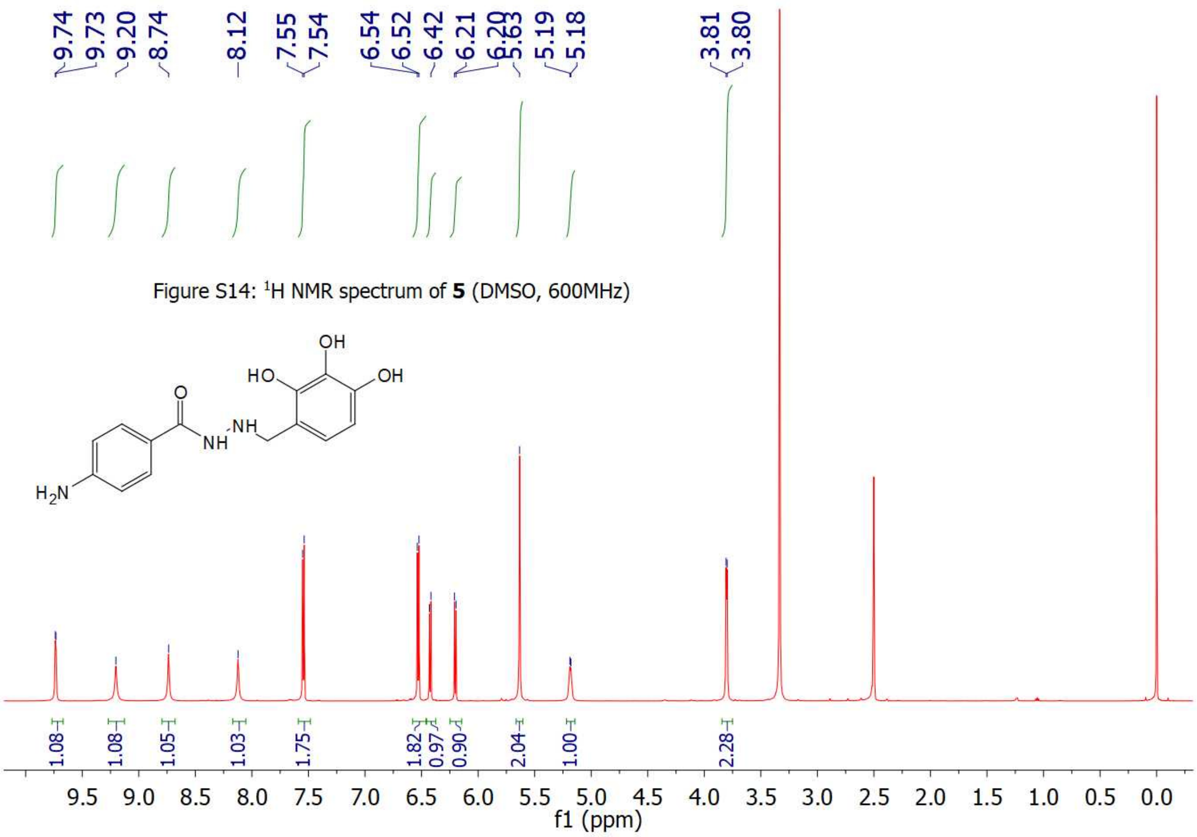

Figure S14: ^{1}H NMR spectrum of **5** (DMSO, 600MHz)

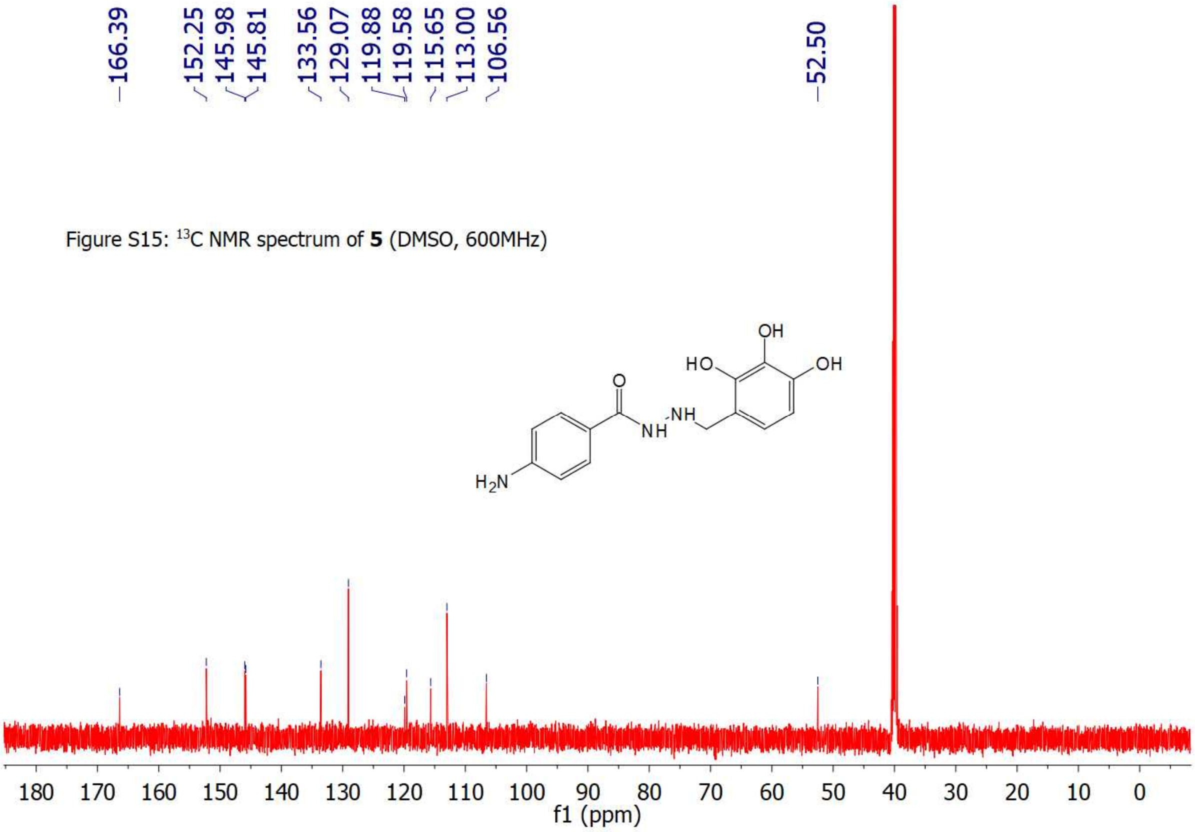

Figure S15: ^{13}C NMR spectrum of **5** (DMSO, 600MHz)

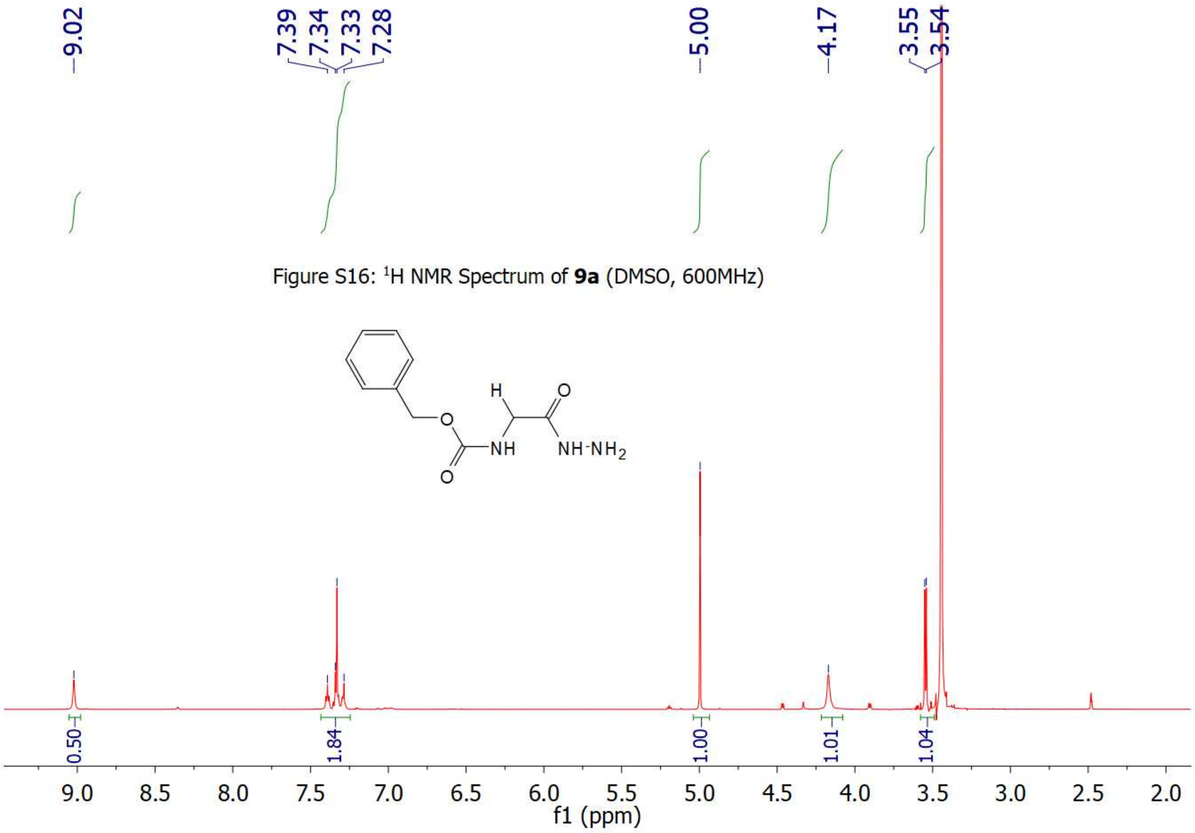

Figure S16: ^{1}H NMR Spectrum of **9a** (DMSO, 600MHz)

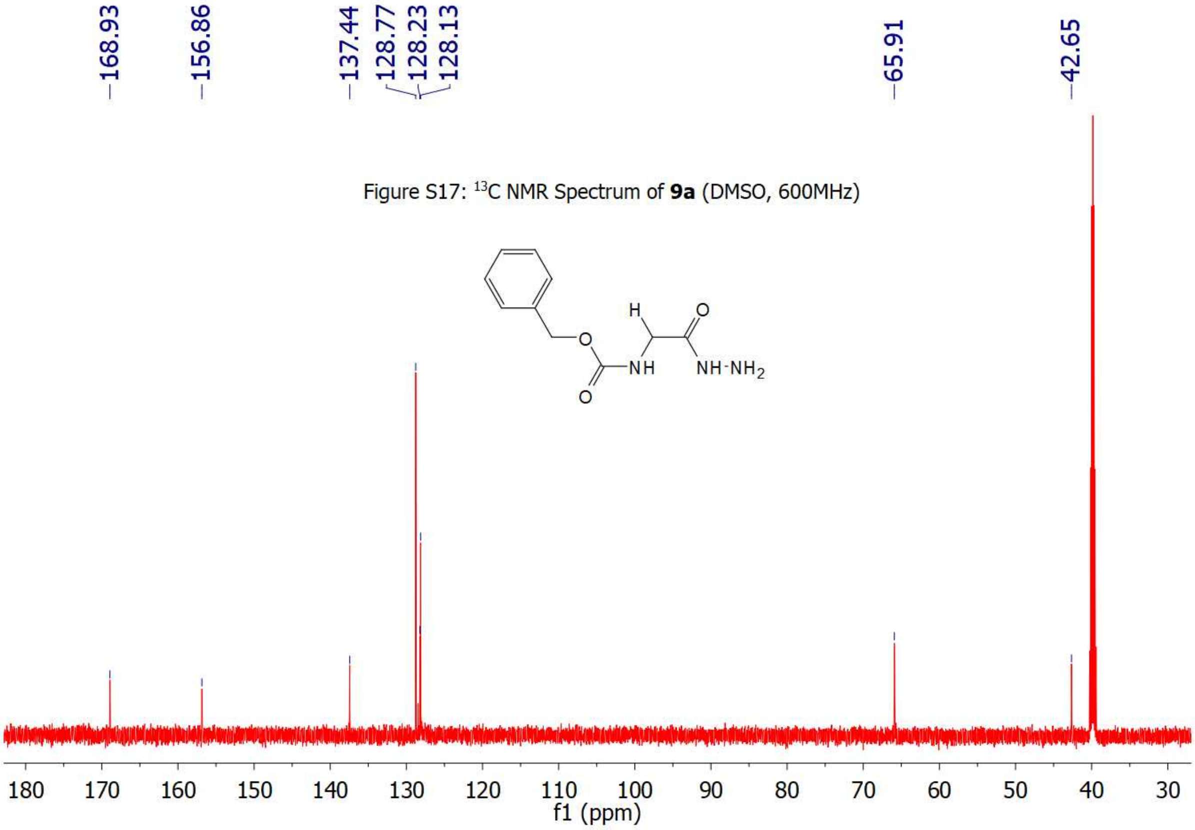

Figure S17: ^{13}C NMR Spectrum of **9a** (DMSO, 600MHz)

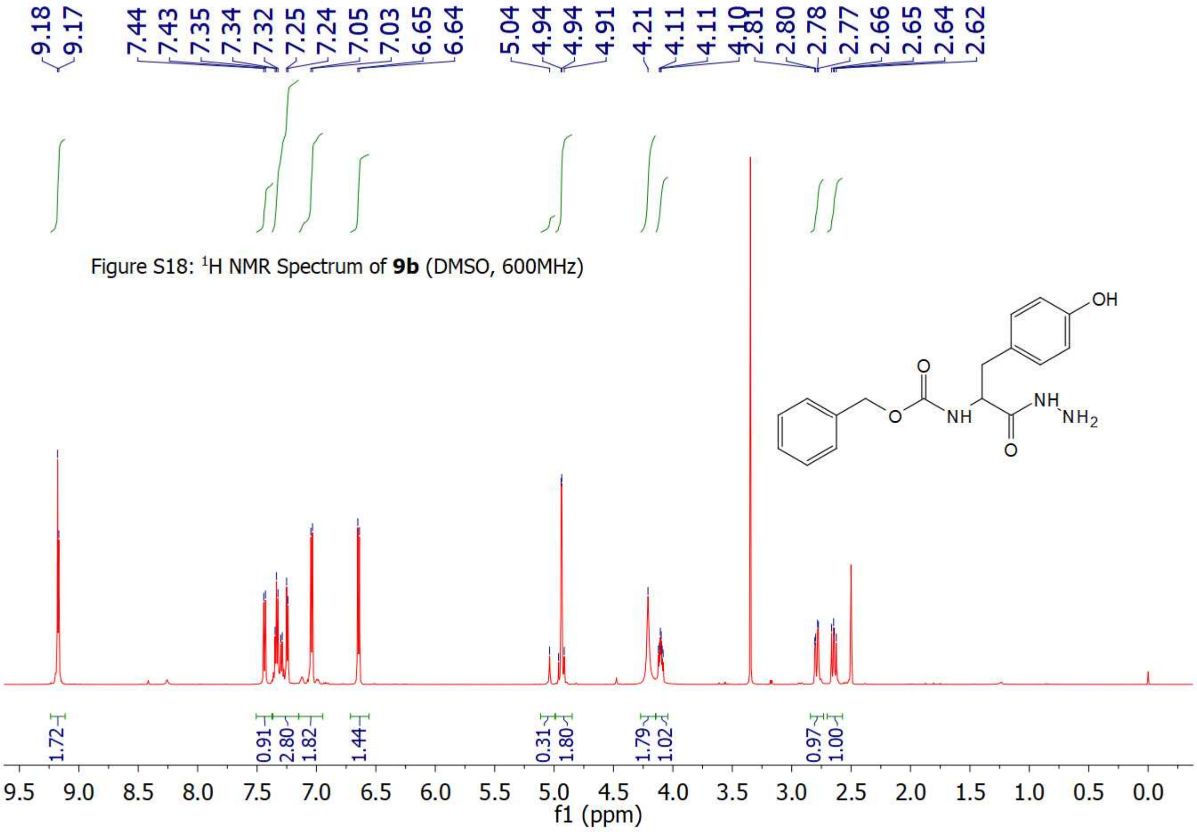

Figure S18: ^{1}H NMR Spectrum of **9b** (DMSO, 600MHz)

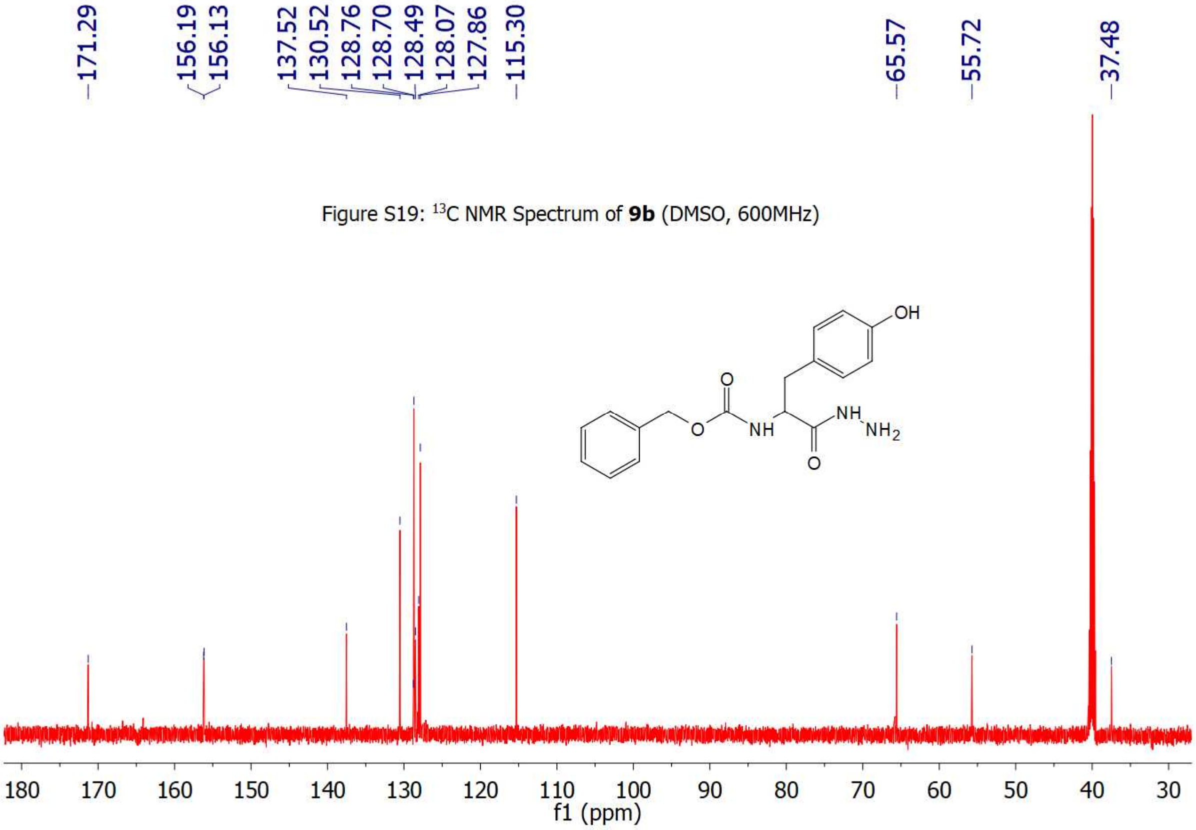

Figure S19: ^{13}C NMR Spectrum of **9b** (DMSO, 600MHz)

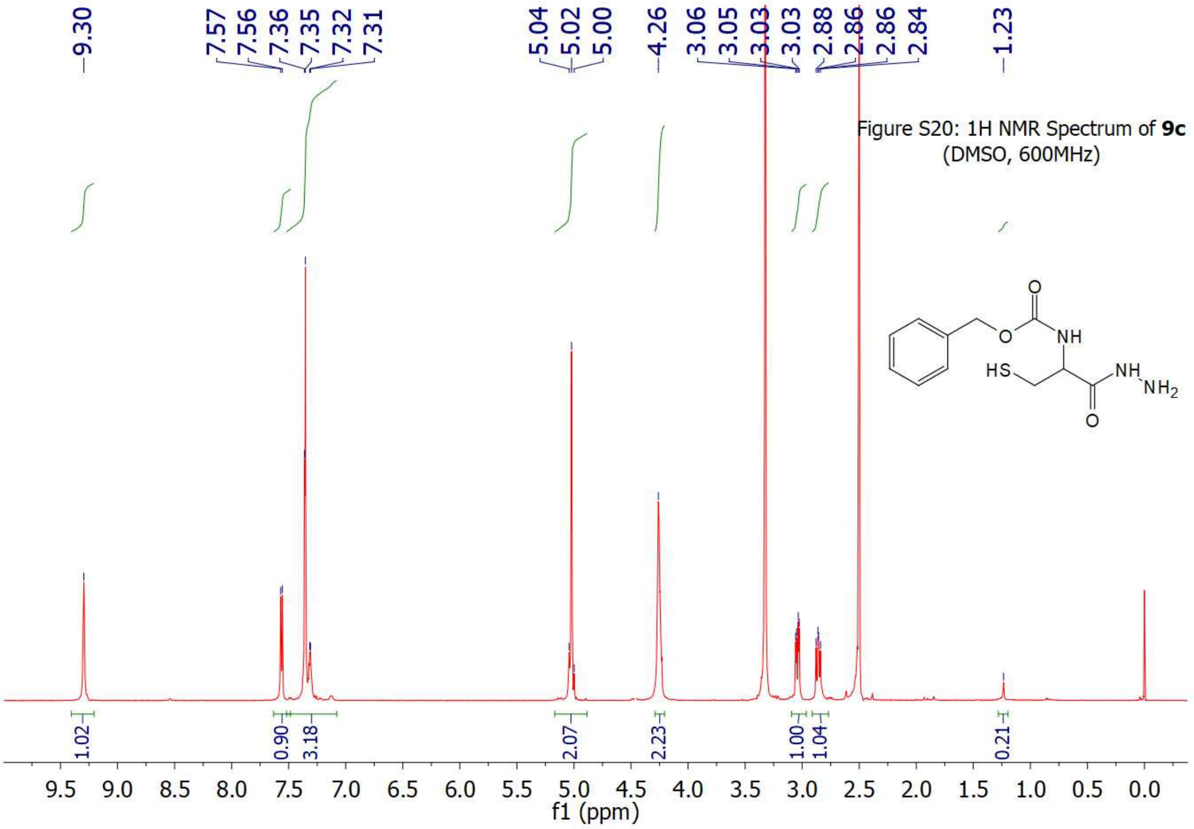

Figure S20: 1H NMR Spectrum of **9c** (DMSO, 600MHz)

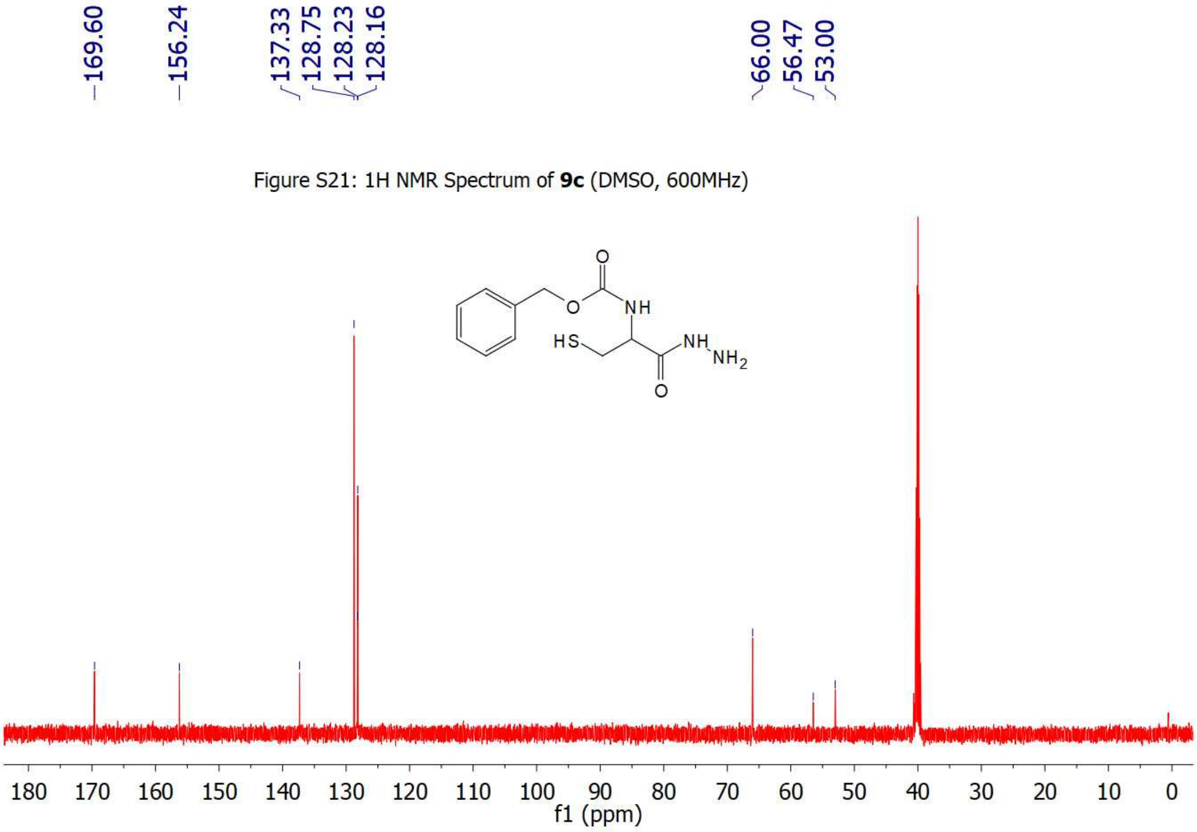

Figure S21: 1H NMR Spectrum of **9c** (DMSO, 600MHz)

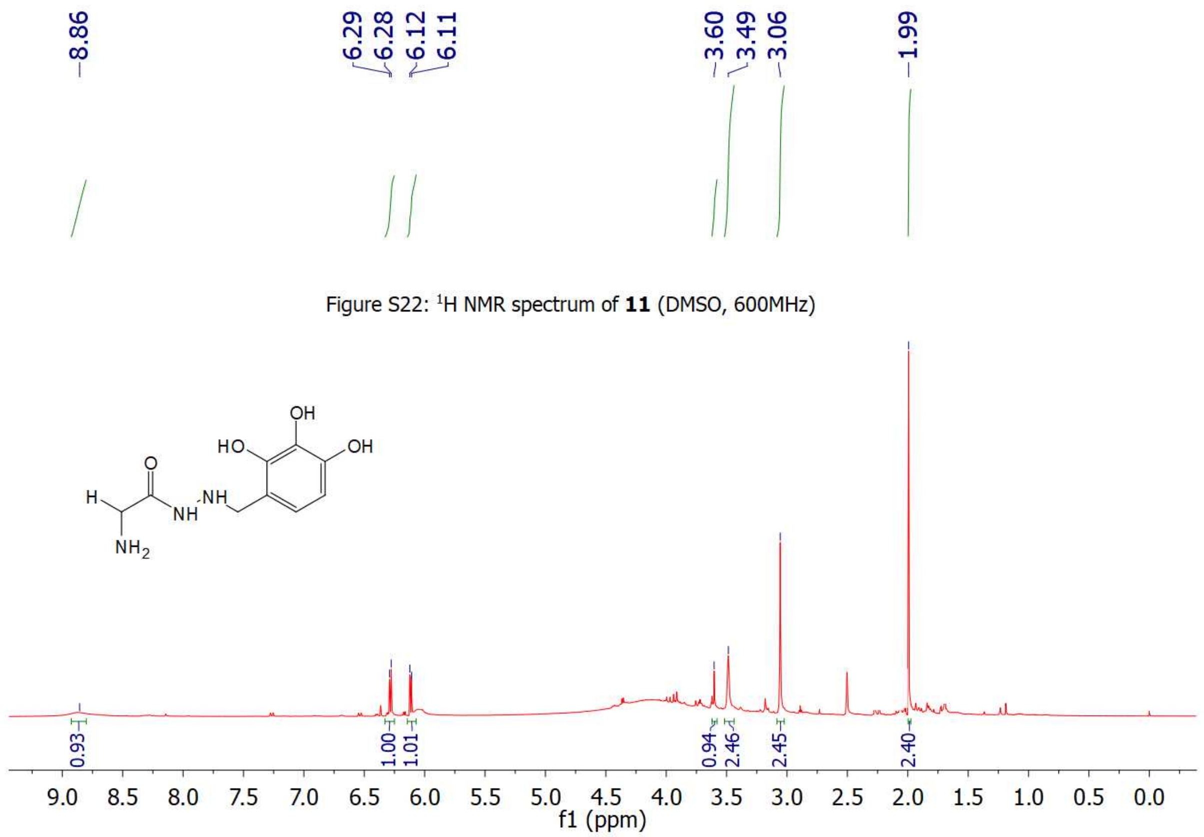

Figure S22: ^{1}H NMR spectrum of **11** (DMSO, 600MHz)

*According to TLC (Thin Layer Chromatography) the compound **11** is pure but probably contains undetectable residual palladium catalyst and water.

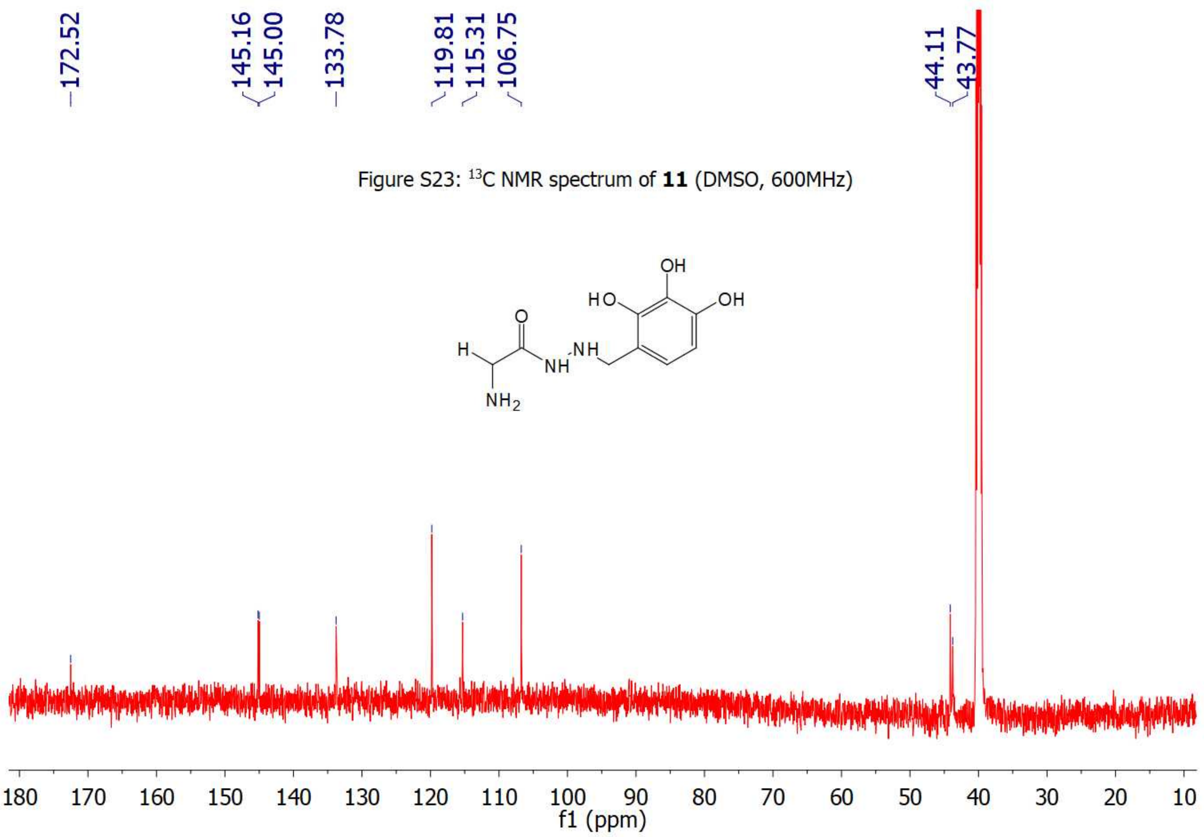

Figure S23: ^{13}C NMR spectrum of **11** (DMSO, 600MHz)

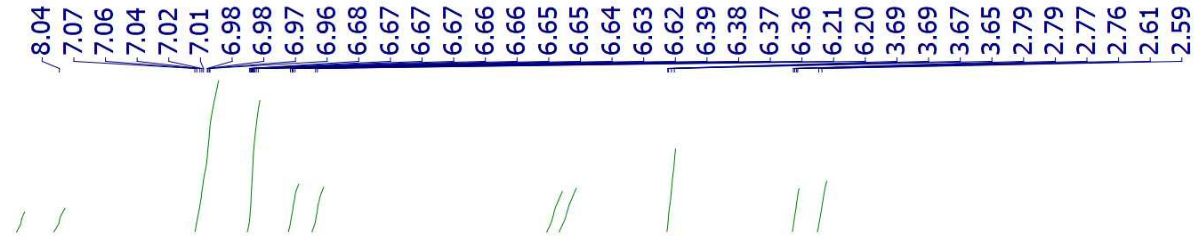

Figure S24: ¹H NMR spectrum of **12** (DMSO, 600MHz)

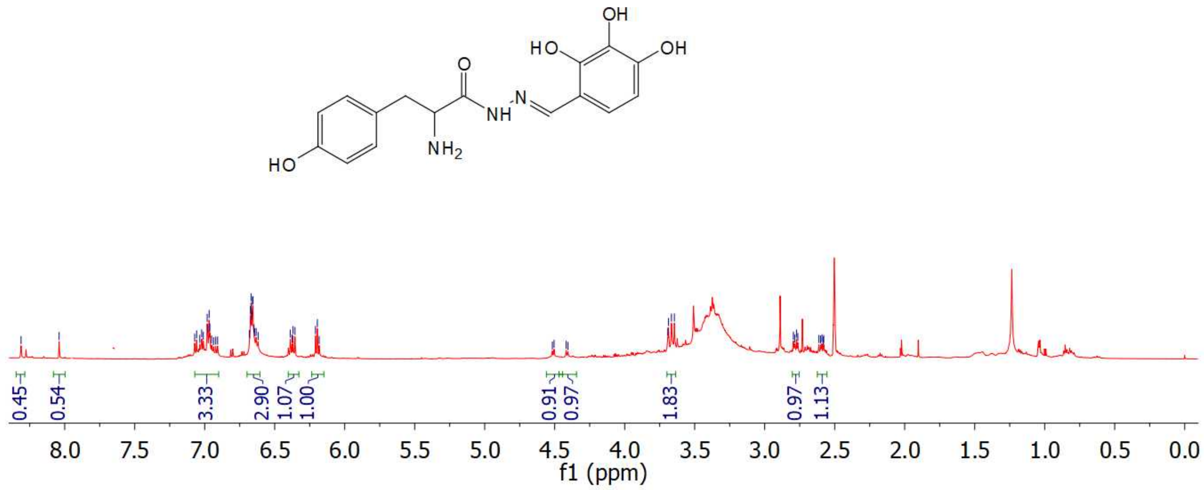

*According to TLC (Thin Layer Chromatography) the compound **12** is pure but probably contains undetectable residual palladium catalyst and water.

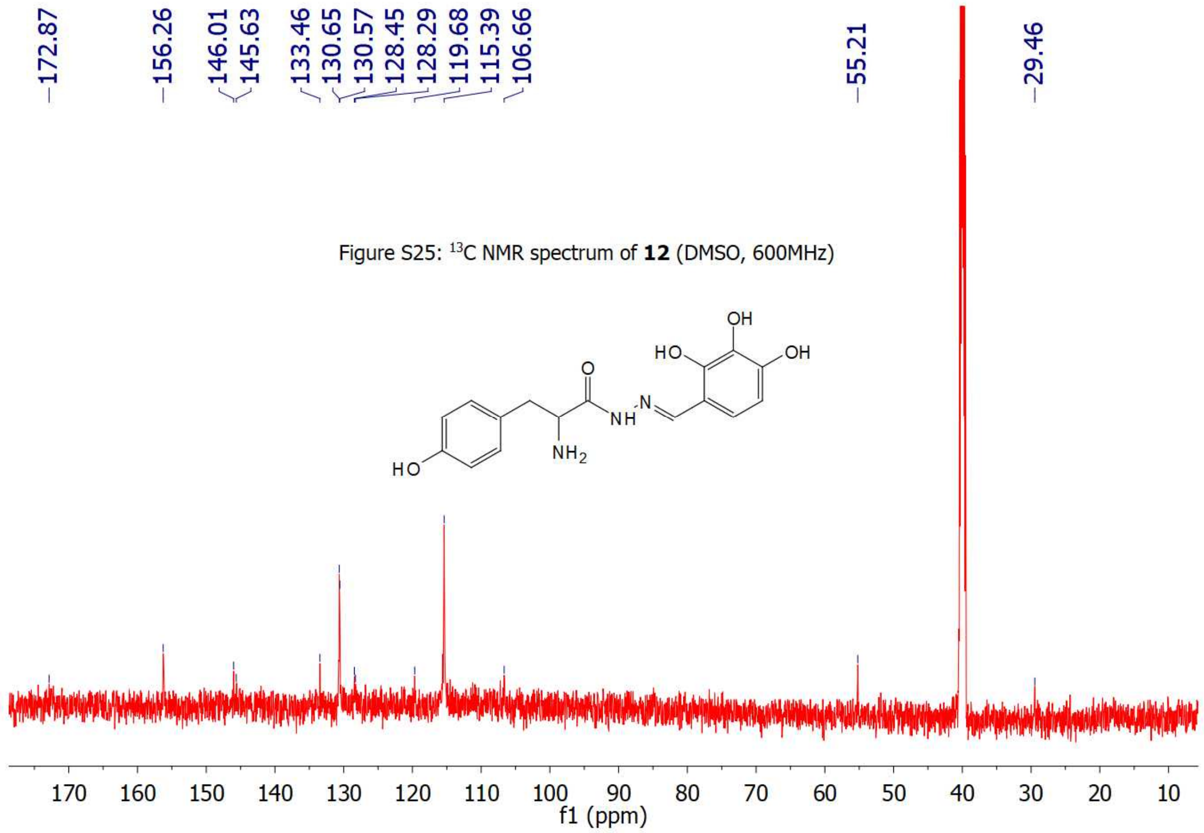

Figure S25: ¹³C NMR spectrum of **12** (DMSO, 600MHz)

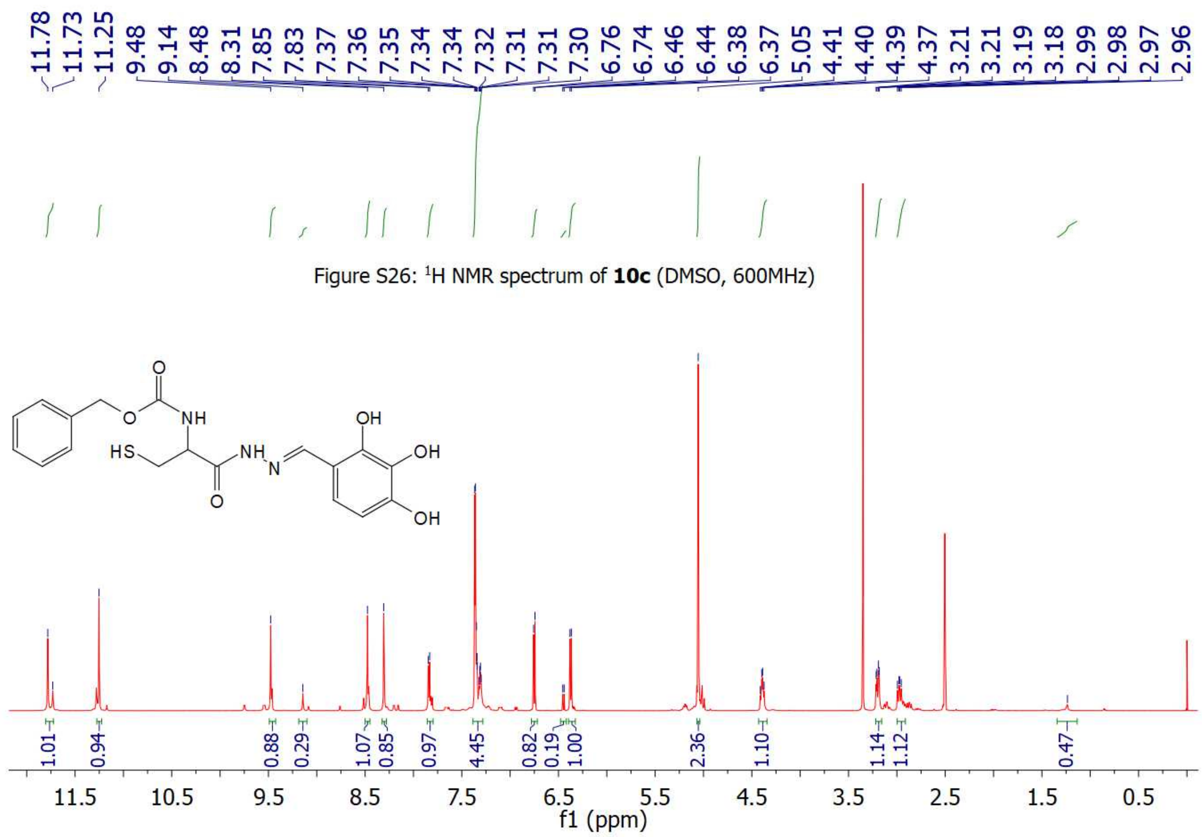

Figure S26: ^{1}H NMR spectrum of **10c** (DMSO, 600MHz)

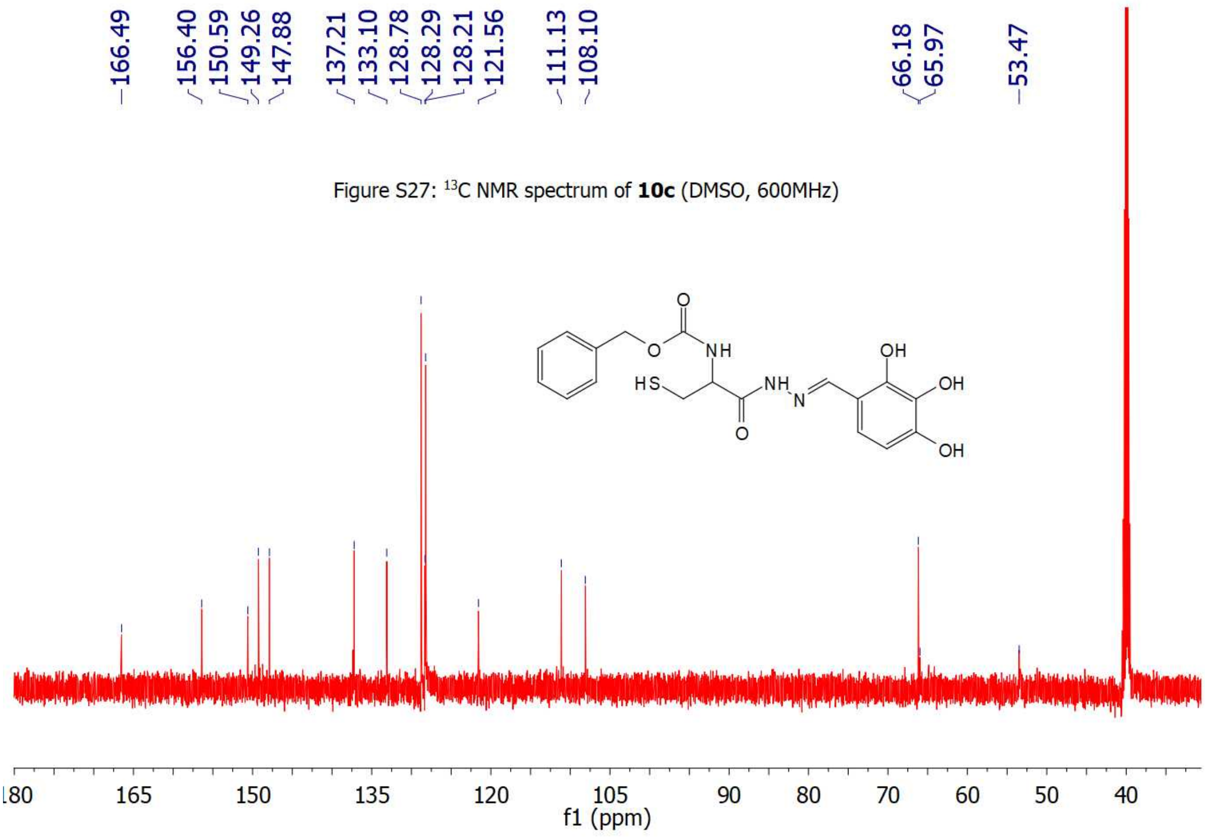

Figure S27: ^{13}C NMR spectrum of **10c** (DMSO, 600MHz)
